# Supplementary material for: 2-Methoxy-4-Vinylphenol as a Biobased Monomer Precursor for Thermoplastics and Thermoset Polymers
Source: Polymers (Basel). 2023 May 2;15(9):2168. doi: 10.3390/polym15092168 (PMC10181207; doi:10.3390/polym15092168)
Supplement: Supplementary file 1 [file polymers-15-02168-s001.zip › polymers-2319455-supplementary.pdf]

Supporting Information

2-Methoxy-4-vinylphenol as a bio-based monomer platform for thermoplastic and thermoset polymers

Alexandros E. Alexakis [1,2] *Thayanithi Ayyachi* [1], *Maryam Mousa* [1], *Peter Olsén* [2,3] *and Eva Malmström* [1,2,*]

AUTHOR AFFILIATION

[1] Division of Coating Technology, Department of Fibre and Polymer Technology, School of Engineering Sci-ences in Chemistry, Biotechnology and Health, KTH Royal Institute of Technology, Teknikringen 56–58, SE-100 44 Stockholm, Sweden

[2] Wallenberg Wood Science Center, Department of Fibre and Polymer Technology, School of Engineering Sciences in Chemistry, Biotechnology and Health, KTH Royal Institute of Technology, Teknikringen 56, SE-100 44 Stockholm, Sweden

[3] Division of Biocomposites, Department of Fibre and Polymer Technology, KTH Royal Institute of Technol-ogy, School of Engineering Sciences in Chemistry, Biotechnology and Health, Teknikringen 56–58, SE-100 44 Stockholm, Sweden

Correspondence: mavem@kth.se

Table of Contents

EXPERIMENTAL SECTION

1. **Materials and methods** 3
2. **Instruments and measurements** 6

PART A

1. **NMR characterization of MVP-derived monomers** 9
2. **FTIR characterization of MVP-derived monomers for thermoplastic applications** 14
3. **Solution polymerization kinetics** 15
4. **FT-IR characterization of solution polymers** 18
5. **FT-IR characterization of emulsion polymers** 18
6. **Emulsion copolymerization** 19
7. **Digital images of the emulsion polymers** 22
8. **DLS results for P(S_{90}-MVP-$AcOMe_5$-MVP-$AcOH_5$)** 22

9. **TGA of solution and emulsion polymers** **23**

10. **DSC traces of the thermoplastic materials** **24**

11. **Glass transition temperature calculation** **26**

PART B

1. **Amounts of reagents for the synthesis of DVB-like monomers** **27**

2. **Amounts of reagents for thermoset synthesis** **27**

3. **NMR characterization of the DVB-like monomers** **28**

4. **FT-IR characterization of DVB-like monomers for thermosetting applications** **33**

5. **FT-IR characterization of DVB-based thermosets** **34**

6. **Digital images of the thermosets** **34**

7. **TGA results of thermosets** **35**

8. **DSC traces of the thermosets** **36**

9. **Crosslinking density calculation** **38**

10. **Chemical stability and polarity of the thermosets** **39**

11. **Environmental impact and comparison** **41**

EXPERIMENTAL SECTION

1. Materials and methods

Materials. Trans-ferulic acid (FA, 99%), sodium hydroxide (NaOH, ≥98%), diethyl ether (Et_2O), potassium carbonate (K_2CO_3, ≥99%), styrene (S, 99%), benzyl bromide (BzBr, 98%), 2,2′-azobisisobutyronitrile (AIBN, 98%), sodium dodecyl sulfate (SDS, ≥99%), potassium persulfate (KPS, 99%), aluminum potassium sulfate dodecahydrate (alum, $AlK(SO_4)_2 \cdot 12H_2O$), dimethyl sulfoxide (DMSO), 1,3-dibromopropane (1,3-DBP, 97%), 1,6-dibromohexane (1,6-DBH, 97%), 1,10-dibromodecane (1,10-DBD, 97%) and (trimethylolpropane tris(3-mercaptopropionate) (3T, ≥95%) were purchased from Sigma Aldrich. Dimethyl sulfoxide-d_6 (DMSO-d_6, 99.9%), chloroform-d ($CDCl_3$, 99.8%), ethanol absolute (EtOH, 99%), dimethyl formamide (DMF), triethylamine (TEA), acetone, magnesium sulfate ($MgSO_4$, 99%), methanol (MeOH), ethyl acetate (EtOAc), acetonitrile (ACN, ≥99.9%), hydrochloric acid (HCl, 37 wt%, AnalaR NORMAPUR®), chloroform ($CHCl_3$, ≥99.8%) and toluene (≥99.9%) were all purchased from VWR. Methyl bromoacetate (MeBrAc, 97%) and tetra-n-butylammonium iodide (TBAI, 98%) were purchased from Lancaster. Lithium hydroxide (LiOH, 98%) was supplied from Merck, 18-crown-6 (99%) from Arcos, and pentaerythritol tetrakis(3-mercaptopropionate) (4T, 95%) by Bruno Bock Chemische Fabrik GmbH & Co. All chemicals described above were used are received without further purification. Deionized water was used throughout this study.

2-Methoxy-4-vinylphenol (MVP)

MVP was synthesized through the decarboxylation of ferulic acid (FA) according to a previously reported procedure [31]. Typically, FA (12 g, 60 mmol, 1 equiv.) was mixed with DMF (15 mL) in a 250 mL round bottom flask equipped with a magnetic stirrer. Once FA was completely dissolved, TEA (13 g, 0.12 mol, 2 equiv.) was added and the flask was sealed with a septum pierced with needles and it was transferred into an oil bath kept at 100 °C. After the reaction was complete (approximately 3 hours), excess of TEA was removed in rotavap (60 °C) and then Et_2O (60 mL) was added. The organic phase was washed with water (3 x 20 mL) and brine (3 x 5 mL) and dried over $MgSO_4$. Finally, the

organic phase was concentrated in rotovap in order to obtain MVP as a dark yellow viscous oil (yield 79%). ^{1}H NMR (400 MHz, DMSO-d_6, Figure S1) δ 9.11 (s, 1H), 7.05 (d, J = 2.1 Hz, 1H), 6.87 (dd, J = 8.1, 2.0 Hz, 1H), 6.78 (d, J = 8.1 Hz, 1H), 6.61 (dd, J = 17.6, 10.9 Hz, 1H), 5.64 (dd, J = 17.6, 1.1 Hz, 1H), 5.07 (dd, J = 10.9, 1.1 Hz, 1H), 3.80 (s, 3H). ^{13}C NMR (101 MHz, $CDCl_3$, Figure S2) δ 146.70, 145.75, 136.75, 130.39, 120.19, 114.46, 111.59, 108.11, 77.37, 56.00.

Methyl 2-(2-methoxy-4-vinylphenoxy)acetate (MVP-AcOMe)

MVP-AcOMe was prepared according to a previously published protocol [56]. MVP (5.0 g, 33 mmol, 1 equiv.) was mixed with acetone (40 mL) in a 250 mL round bottom flask under stirring, followed by the addition of K_2CO_3 (18.4 g, 133 mmol, 4 equiv.). MeBrAc (6.0 g, 39 mmol, 1.18 equiv.) was added dropwise and the flask was immersed in an oil bath and left for stirring under reflux overnight. The crude reaction mixture was filtered and acetone was removed under reduced pressure. Et_2O (25 mL) was added to the concentrate and subsequently washed with water (3 x 8 mL). The organic phase was dried over $MgSO_4$ and concentrated in rotavap to obtain a dark yellow viscous oil (yield 70%). ^{1}H NMR (400 MHz, DMSO-d_6, Figure S4) δ 7.12 (d, J = 2.1 Hz, 1H), 6.94 (dd, J = 8.3, 2.0 Hz, 1H), 6.85 (d, J = 8.3 Hz, 1H), 6.65 (dd, J = 17.7, 10.9 Hz, 1H), 5.73 (d, J = 16.5 Hz, 1H), 5.17 (s, 1H), 4.78 (s, 2H), 3.81 (s, 3H), 3.69 (s, 3H). ^{13}C NMR (101 MHz, $CDCl_3$, Figure S5) δ 169.54, 149.74, 147.18, 136.43, 132.53, 119.31, 114.14, 112.73, 109.51, 66.57, 56.02, 52.38.

2-(2-Methoxy-4-vinylphenoxy)acetic acid (MVP-AcOH)

MVP-AcOH was synthesized from MVP-AcOMe (above) without prior purification, hence the amounts of the reagents described below were based on 5.0 g (33 mmol) of MVP [56]. The concentrated crude product was mixed with an aqueous mixture of MeOH (150 mL, H_2O:MeOH 1:1) and LiOH (9.8 g, 0.4 mol, 8 equiv.) was added, while cooling the sample in an ice bath. The reaction was left under stirring at room temperature overnight. Thereafter, the solution was concentrated in a rotavap and 10% HCl was added until the pH was between 1-2. The product was extracted from the aqueous phase with EtOAc (3 x 280 mL). The organic phase was washed with water, dried over $MgSO_4$ and concentrated in rotavap. The product was collected as whitish solids and dried under vacuum overnight (yield 74.6%). ^{1}H NMR

(400 MHz, DMSO-d_6, Figure S6) δ 12.99 (s, 1H), 7.12 (d, J = 2.0 Hz, 1H), 6.94 (dd, J = 8.3, 2.0 Hz, 1H), 6.82 (d, J = 8.3 Hz, 1H), 6.65 (dd, J = 17.6, 10.9 Hz, 1H), 5.72 (dd, J = 17.6, 1.0 Hz, 1H), 5.15 (dd, J = 10.8, 1.0 Hz, 1H), 4.67 (s, 2H), 3.81 (s, 3H). ^{13}C NMR (101 MHz, DMSO-d_6, Figure S7) δ 170.16, 149.02, 147.23, 136.37, 130.92, 119.08, 113.16, 112.38, 109.64, 65.10, 55.61.

Synthesis of divinyl benzene (DVB)-like monomers based on MVP

All DVB-like monomers were prepared using the same protocol, in accordance to a previously published work with some modifications [57]. The case of 1,3-bis(2-methoxy-4-vinylphenoxy)propane (1,3-MVP) is described. MVP (6.5 g, 43 mmol, 1 equiv.) was mixed with acetone (160 mL, 50 equiv.) in a 250 mL two- or three-necked round bottom flask equipped with a stirrer. Afterwards, K_2CO_3 (23.9 g, 173 mmol, 4 equiv.) and TBAI (0.24 g, 0.65 mmol, 0.015 equiv.) were added and the mixture was stirred for an hour. 1,3-dibromopropane (1,3-DBP) (2.0 mL, 20 mmol, 0.45 equiv.) was then added dropwise and the reaction mixture was stirred for 15 min. A condenser was attached and the flask was immersed in a preheated oil bath and reacted under reflux (56 °C) overnight. The reaction was followed by TLC. After the disappearance of the reagents (followed by TLC), the solids were filtered off and the filtrate was concentrated in the rotavap. Then, it was washed twice with EtOAc and thrice with 1M NaOH and finally once with brine. The organic phase was dried over $MgSO_4$ and concentrated in rotavap to obtained the crude 1,3-MVP as yellow solids (yield 85%). Finally, the product was triturated with EtOH to obtain pure 1,3-MVP. ^{1}H NMR (400 MHz, $CDCl_3$, Figure S23) δ 6.99 (d, J = 1.9 Hz, 2H), 6.95 (dd, J = 8.3, 1.9 Hz, 2H), 6.90 (d, J = 8.2 Hz, 2H), 6.67 (dd, J = 17.5, 10.9 Hz, 2H), 5.63 (dd, J = 17.6, 1.0 Hz, 2H), 5.17 (dd, J = 10.9, 0.9 Hz, 2H), 4.27 (t, J = 6.2 Hz, 4H), 3.89 (s, 6H), 2.38 (p, J = 6.2 Hz, 2H). ^{13}C NMR (101 MHz, $CDCl_3$, Figure S24) δ 149.64, 148.48, 136.64, 131.14, 119.59, 113.33, 112.00, 109.27, 65.94, 56.06, 29.37. Its 2D HSQC NMR can be found in Figure S25.

1,6-Bis(2-methoxy-4-vinylphenoxy)hexane (1,6-MVP) and 1,10-bis(2-methoxy-4-vinylphenoxy)decane (1,10-MVP) were obtained as white solids after purification (yields 71% and 83%, respectively). Analytical amounts of each reagent used in the synthesis of the crosslinkers can be found in Table S5.

1,6-MVP: ^{1}H NMR (400 MHz, $CDCl_3$, Figure S26) δ 6.99 (d, J = 2.0 Hz, 2H), 6.95 (dd, J = 8.3, 2.0 Hz, 2H), 6.84 (d, J = 8.2 Hz, 2H), 6.67 (dd, J = 17.5, 10.9 Hz, 2H), 5.63 (dd, J = 17.5, 0.9 Hz, 2H), 5.17 (dd, J = 10.8, 0.8 Hz, 2H), 4.05 (t, J = 6.8 Hz, 4H), 3.91 (s, 6H), 1.95 – 1.82 (m, 4H), 1.58 (s, 8H). ^{13}C NMR (101 MHz, $CDCl_3$, Figure S27) δ 149.56, 148.65, 136.67, 130.84, 119.58, 112.88, 111.86, 109.19, 69.03, 56.09, 29.25, 25.93. Its 2D HSQC NMR can be found in Figure S28.

1,10-MVP: ^{1}H NMR (400 MHz, $CDCl_3$, Figure S29) δ 6.99 (d, J = 1.9 Hz, 2H), 6.95 (dd, J = 8.2, 2.0 Hz, 2H), 6.85 (d, J = 8.2 Hz, 2H), 6.67 (dd, J = 17.5, 10.8 Hz, 2H), 5.63 (dd, J = 17.6, 0.9 Hz, 2H), 5.16 (dd, J = 10.8, 0.9 Hz, 2H), 4.03 (t, J = 6.9 Hz, 4H), 3.91 (s, 6H), 1.87 (q, J = 7.2 Hz, 4H), 1.53 – 1.29 (m, 14H). ^{13}C NMR (101 MHz, $CDCl_3$, Figure S30) δ 149.50, 148.67, 136.65, 130.72, 119.55, 112.77, 111.77, 109.05, 69.14, 56.05, 29.57, 29.48, 29.25, 26.04. Its 2D HSQC NMR can be found in Figure S31.

2. Instruments and measurements

Nuclear magnetic resonance (NMR). ^{1}H-, ^{13}C- and 2D-NMR (HSQC) were obtained from a Bruker Avance 400 spectrometer (^{1}H: 400.13 MHz; ^{13}C: 100.62 MHz) equipped with a 5 mm broad-band multinuclear (PABBO) probe at 25 °C using Bruker TopSpin v2.1 software. The number of scans were changed for ^{13}C- and 2D-NMR (512 and 32, respectively). The samples were dissolved either in DMSO-d_6 or $CDCl_3$.

Differential scanning calorimetry (DSC). A Mettler-Toledo DSC 1 instrument system was used to measure the thermal properties of the polymers and monomers. Approximately 5-8 mg of sample was used and measurements were performed under nitrogen flow (50 mL/min) from −50 to +200 °C with a heating rate of 10 °C/min. The melting temperature (T_m) and enthalpy ($\Delta H_{melting}$) were obtained from the first heating cycle whereas the glass transition temperature (T_g) values have been reported from the second heating cycle. Duplicate measurements were obtained for every sample and the average values have been reported.

Thermogravimetric analysis (TGA). The thermal stability of the samples was evaluated thermogravimetrically with a Mettler Toledo TGA/DSC 1 instrument. Approximately 7-10 mg of the

samples were placed in 70 μL alumina crucibles heated from 30 to 600 °C with a 10 °C/min heating ramp under nitrogen flow (50 mL/min). Duplicate measurements were obtained for every sample and the average values have been reported.

Size exclusion chromatography (SEC). The molecular weight and polydispersity of the synthesized polymers was evaluated by a TOSOH EcoSEC HLC-8320GPC system equipped with an EcoSEC RI detector and three columns (PSS PFG 5 μm; Microguard, 100 Å, and 300 Å; MW resolving range: 100-300,000 g/mol) from PSS GmbH, using DMF as solvent with 0.01 M LiBr as the mobile phase at 50 °C with a flow rate of 0.2 mL/min. A PMMA calibration standards wwere used ranging from 700 to 2,000,000 g/mol.

Dynamic light scattering (DLS). The hydrodynamic diameter (D_H), given as Z-average, polydispersity index (PdI) and electrophoretic mobility (ζ) of the latexes were determined using a Malvern Zetasizer NanoZS at 25 °C. Each value used in this study is averaged over ten measurements which were averaged over three consecutive runs. For all measurements, the concentration of the latex dispersions was 0.1 wt% and the solvent was deionized water. The standard used for the size correlation was PS latex.

Fourier transform infrared spectroscopy (FT-IR). A PerkinElmer Spectrum 100 instrument equipped with a diamond crystal and an MKII Golden Gate accessory (Specac Ltd.) was used to acquire the FT-IR spectra of the different monomers, polymers and thermoset films. The spectra were obtained at room temperature in attenuated total reflection (ATR) mode between 600 cm^{-1} and 4000 cm^{-1}, and each measurement was averaged over 16 scans with a resolution of 4 cm^{-1}. All spectra were baseline corrected and normalized to 1600 cm^{-1} signal via PerkinElmer Spectrum software V10.5.1.

Dynamical mechanical analysis (DMA). The viscoelastic properties of the thermosets were obtained by a TA Instruments DMA Q800 equipped with a gas cooling accessory and tensile film clamps. The samples with approximate dimensions of 10.6 mm × 5.3 mm × 1.1 mm were subjected to a strain of 0.1% at 1 Hz with a preload force of 0.01 N (force track 125%). Initially, the samples were cooled to -50 °C and equilibrated for 5 min. Then, they were heated to 250 °C with a heating rate of 3 °C/min. The values reported of storage modulus (E′), loss modulus (E″), and $tan\delta$ (ratio between E″ and E′)

were averaged over 3 measurements. The glass transition temperature was determined from the maximum of $tan\delta$ ($T_g^{tan\delta}$).

Contact angle measurement (CA). The CA of each thermosetting film against water was monitored by using Theta Lite optical tensiometer coupled with a remote-controlled syringe (Biolin Scientific). All samples were kept for at least 24 h at 50% RH at ambient temperature before the measurement. The sessile drop method with a volume of 4 μL was used. Images of the deposited droplet were captured every 25 ms and evaluated using the OneAttention software (Biolin Scientific) to obtain the average contact angle. Each sample was measured in three distinct positions to increase statistics.

PART A

1. NMR characterization of MVP-derived monomers

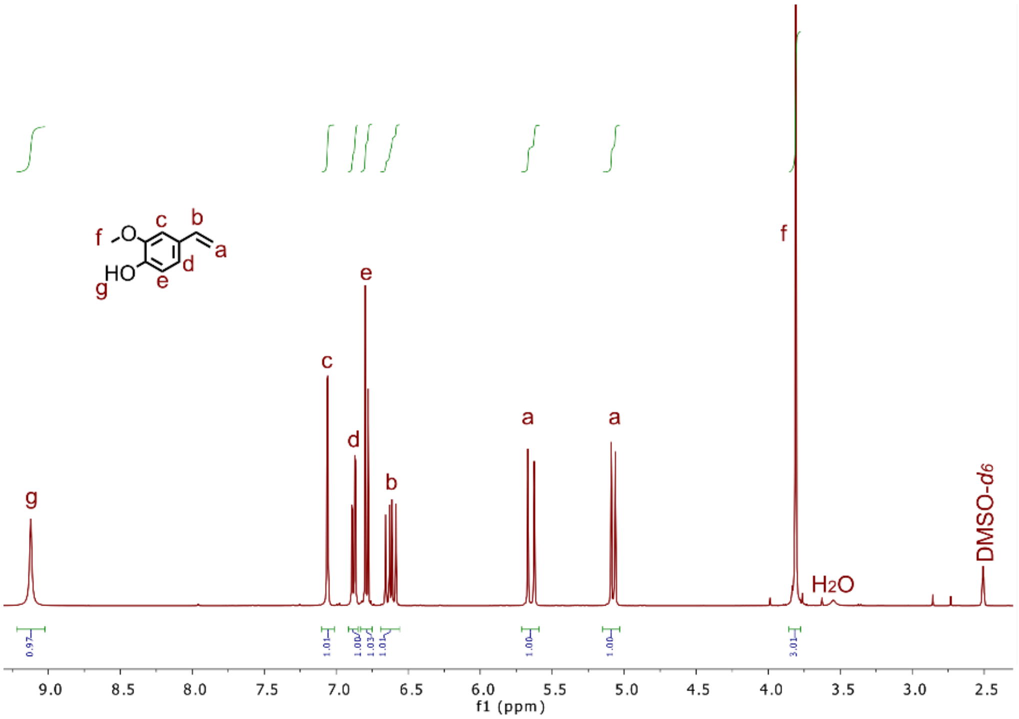

Figure S1. *^{1}H-NMR of MVP in DMSO-d_6.*

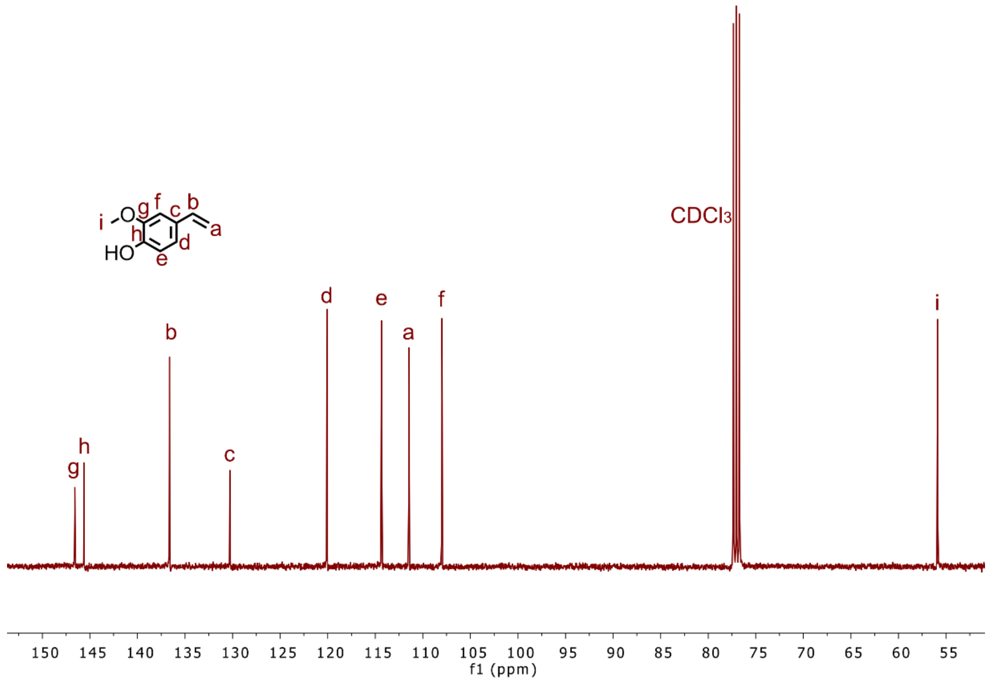

Figure S2. *^{13}C-NMR of MVP in $CDCl_3$.*

The stability of MVP was monitored with ^{1}H-NMR when it was stored in the fridge under argon atmosphere for 20 days (Figure S3). The less defined splitting of the aromatic region can be seen at day 20, which was hypothesized to be the maximum shelf-life of MVP.

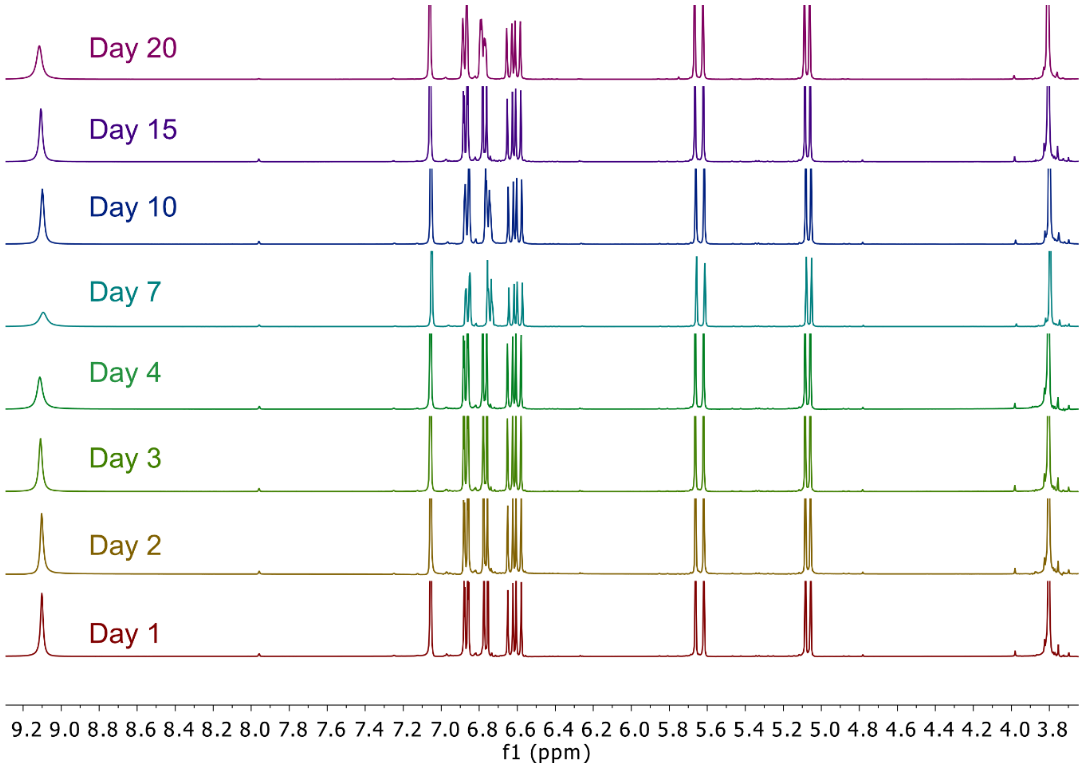

Figure S3. *^{1}H-NMR of MVP over the range of 20 days stored in the fridge under argon.*

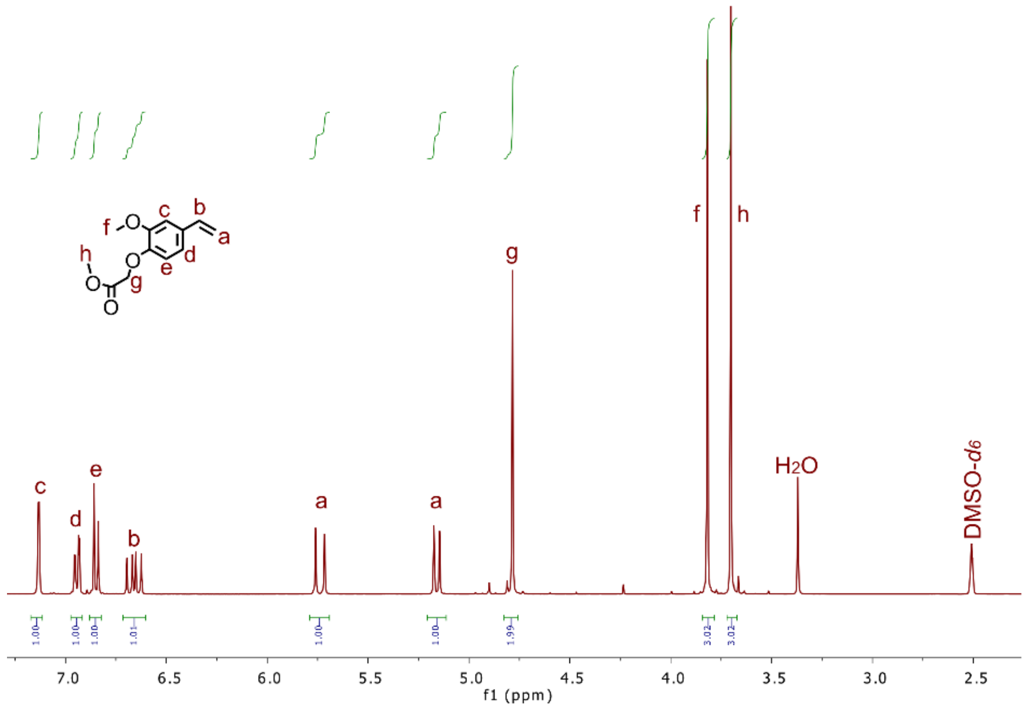

Figure S4. *^{1}H-NMR of MVP-AcOMe in DMSO-d$_6$.*

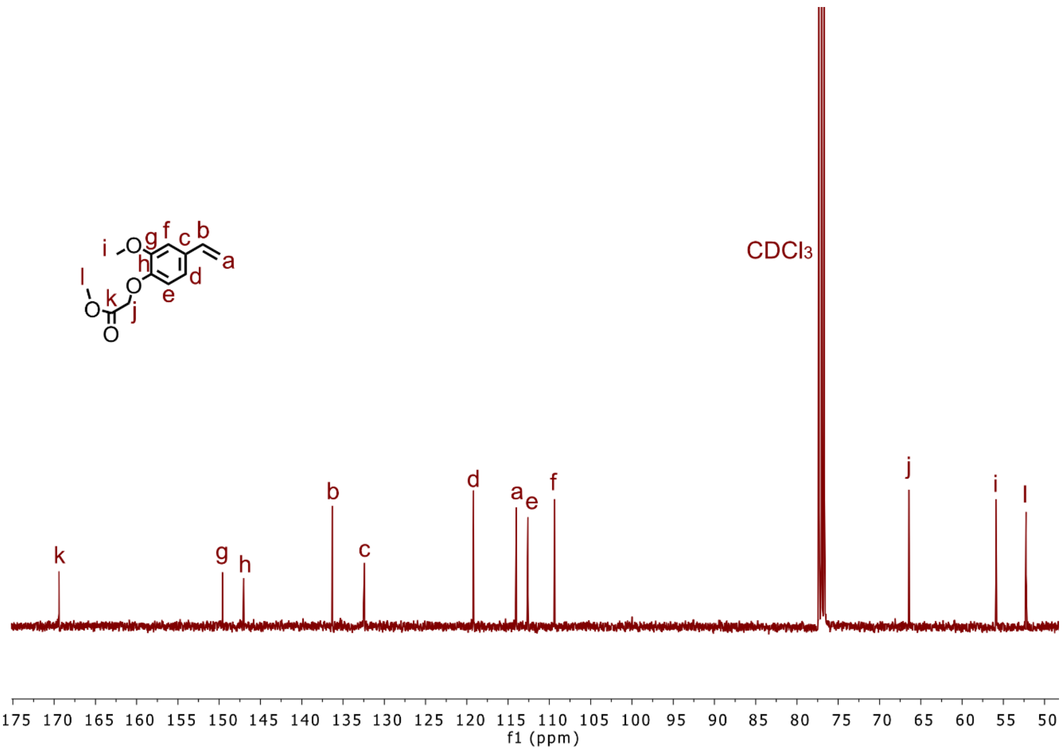

Figure S5. *^{13}C-NMR of MVP-AcOMe in CDCl$_3$.*

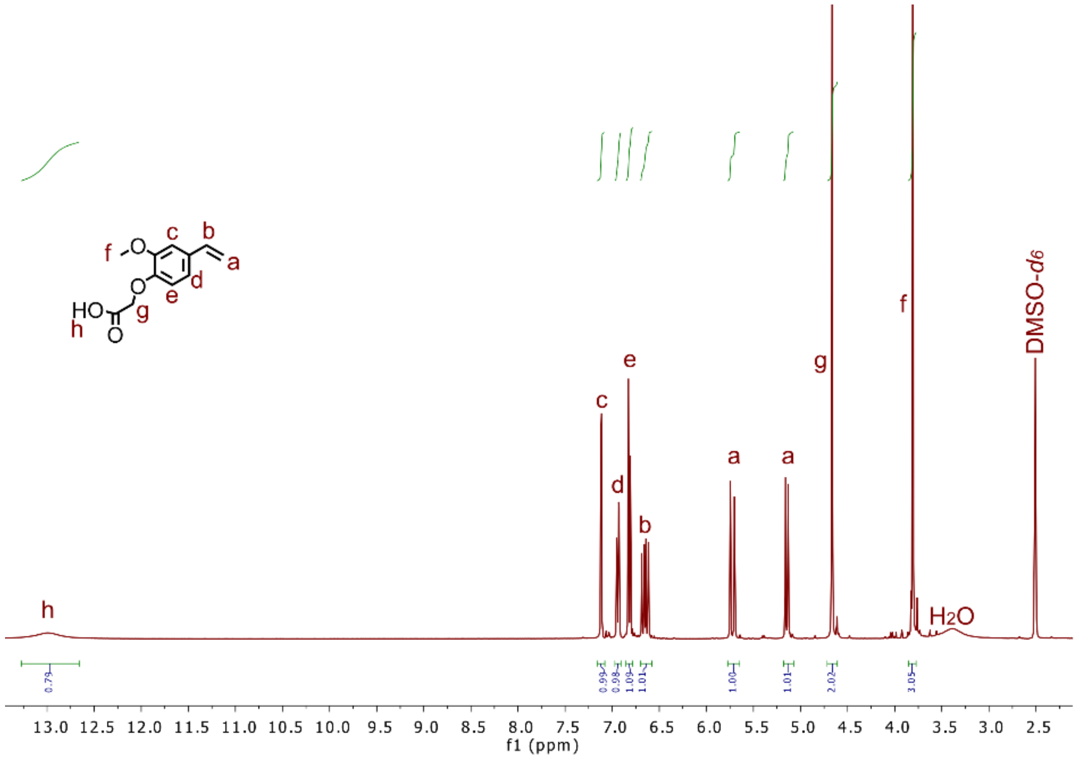

Figure S6. *^{1}H-NMR of MVP-AcOH in DMSO-d6.*

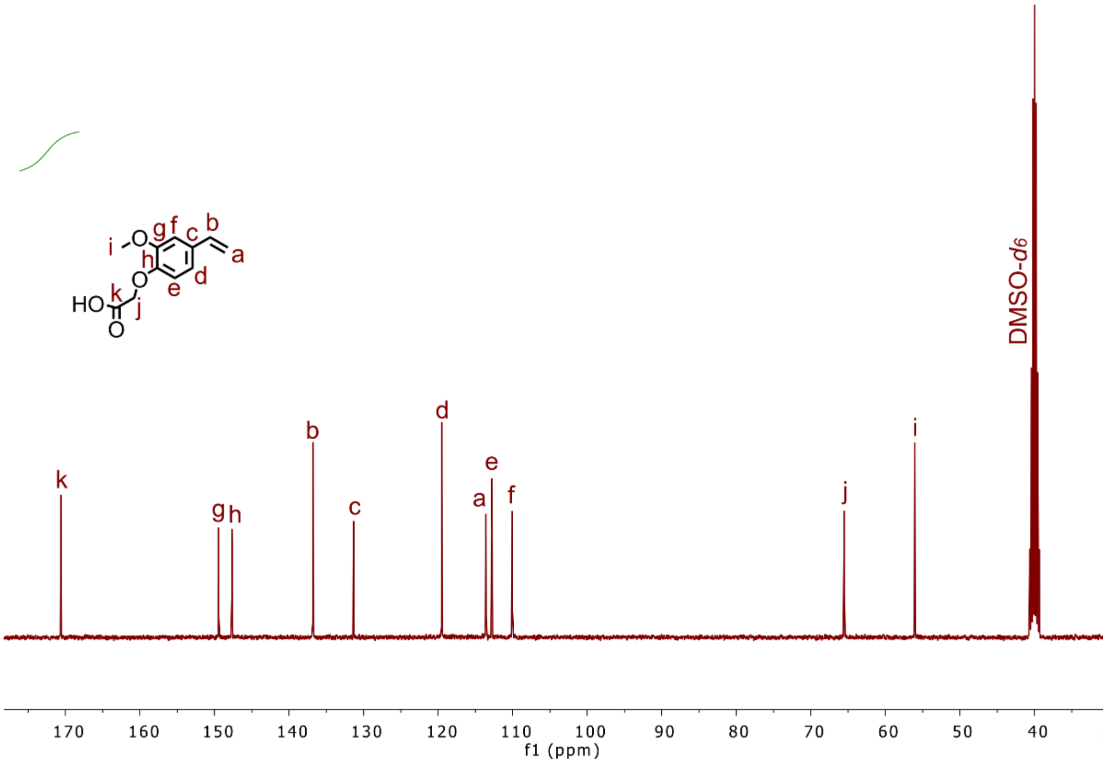

Figure S7. *^{13}C-NMR of MVP-AcOH in DMSO-d6.*

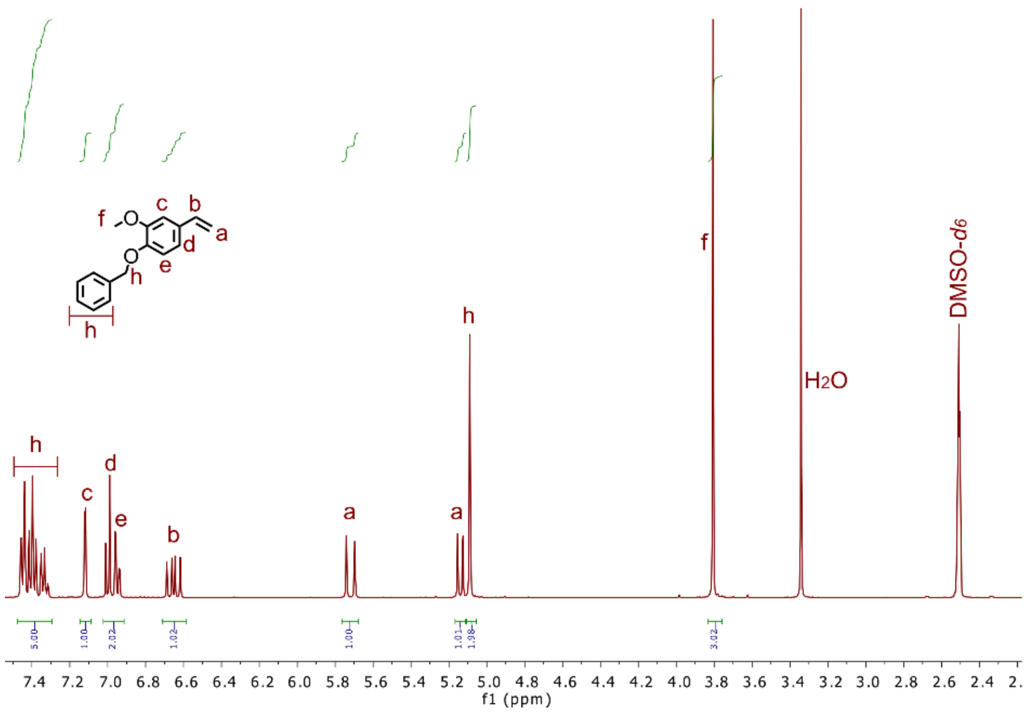

Figure S8. *^{1}H-NMR of MVP-Bz in DMSO-d6.*

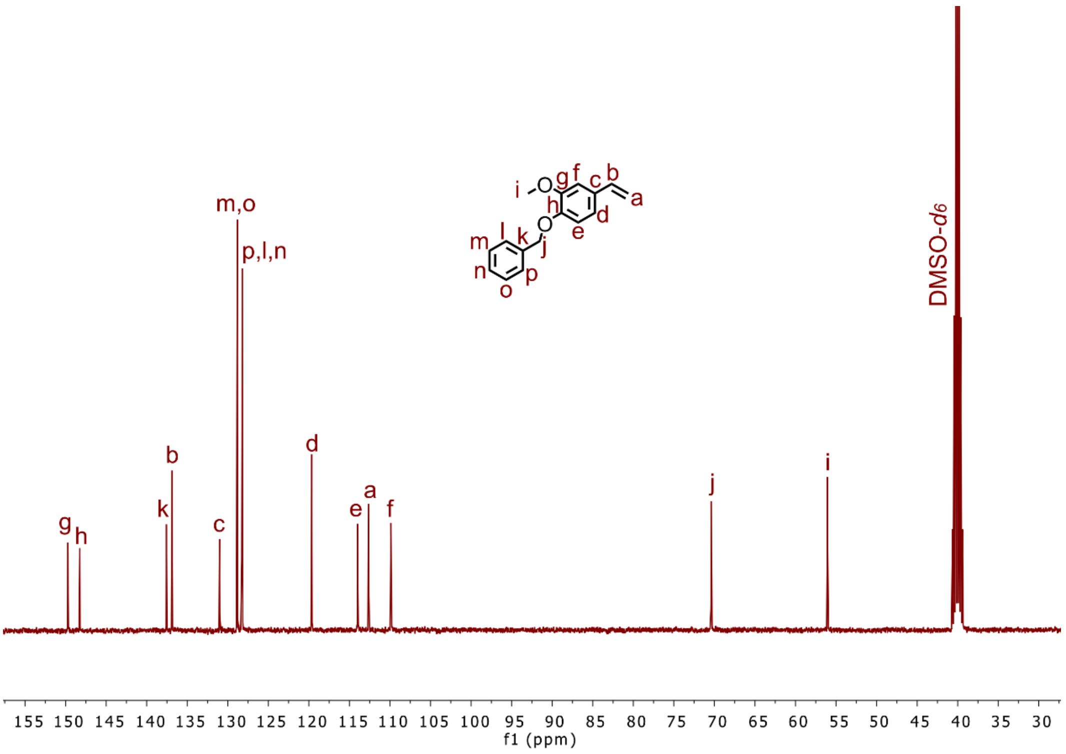

Figure S9. *^{13}C-NMR of MVP-Bz in DMSO-d6.*

2. FT-IR characterization of MVP-derived monomers for thermoplastic applications

The chemical composition of the bio-based monomers derived from MVP was also verified by FT-IR (Figure S10). MVP is characterized by the broad phenolic peak at around 3500 cm^{-1}, whereas the rest of the monomers do not exhibit this peak which indicates the successful modification. Additionally, MVP-AcOMe and MVP-AcOH exhibit a carbonyl peak at 1735-1754 cm^{-1} (marked with the dashed rectangle) and the latter also has a broader peak between 2500 and 3100 cm^{-1} due to its carboxylic acid functionality. Finally, MVP-Bz has a similar pattern as MVP-AcOMe, but has a more pronounced peaks related to aromatics (smeared out due to normalization).

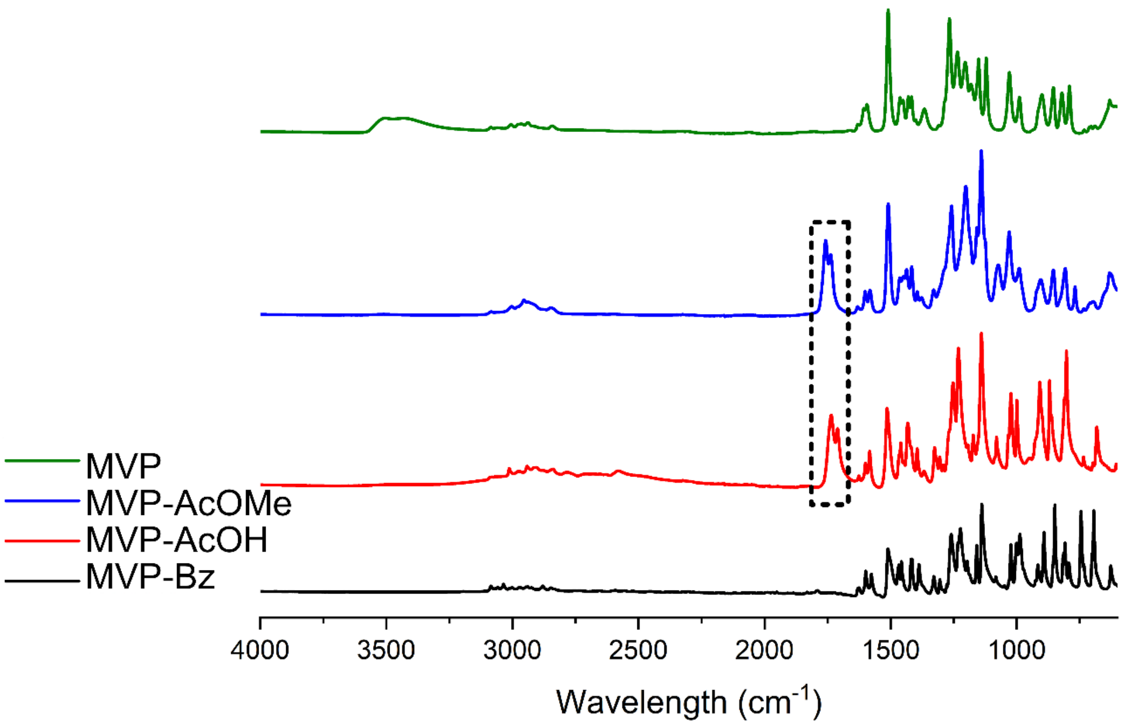

Figure S10. *FT-IR of bio-based monomers derived from MVP.*

3. Solution polymerization kinetics

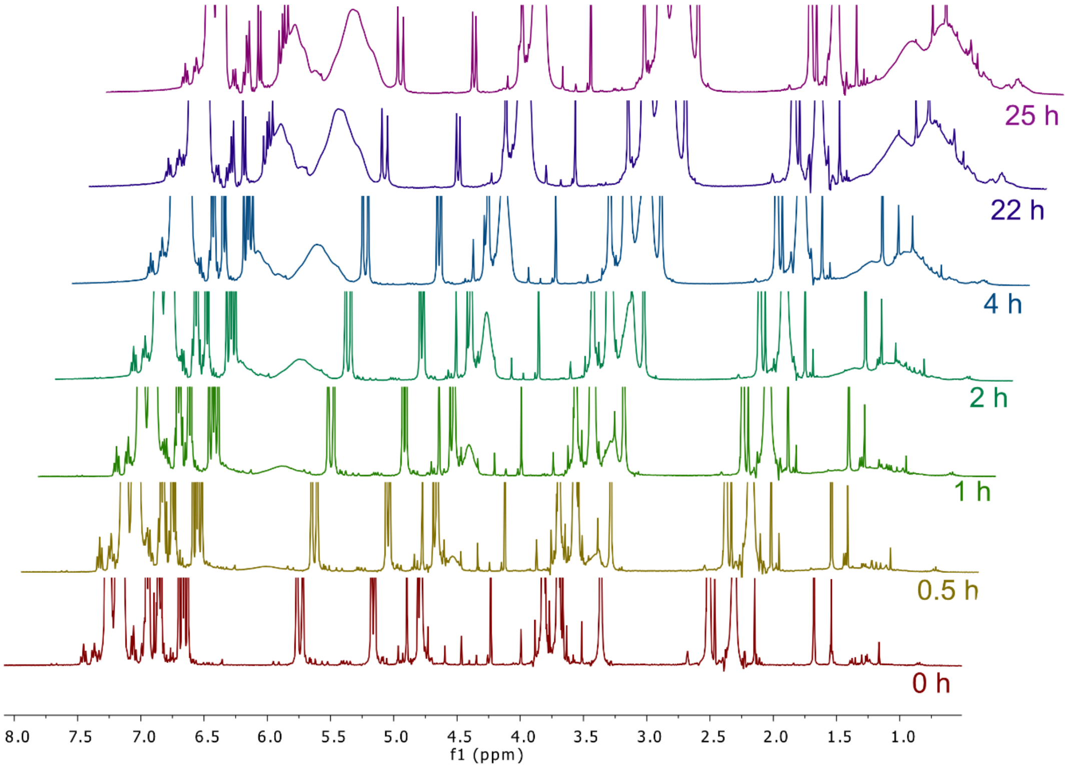

Figure S11. *^{1}H-NMR kinetics of P(MVP-AcOMe) prepared by solution polymerization in DMSO-d$_6$.*

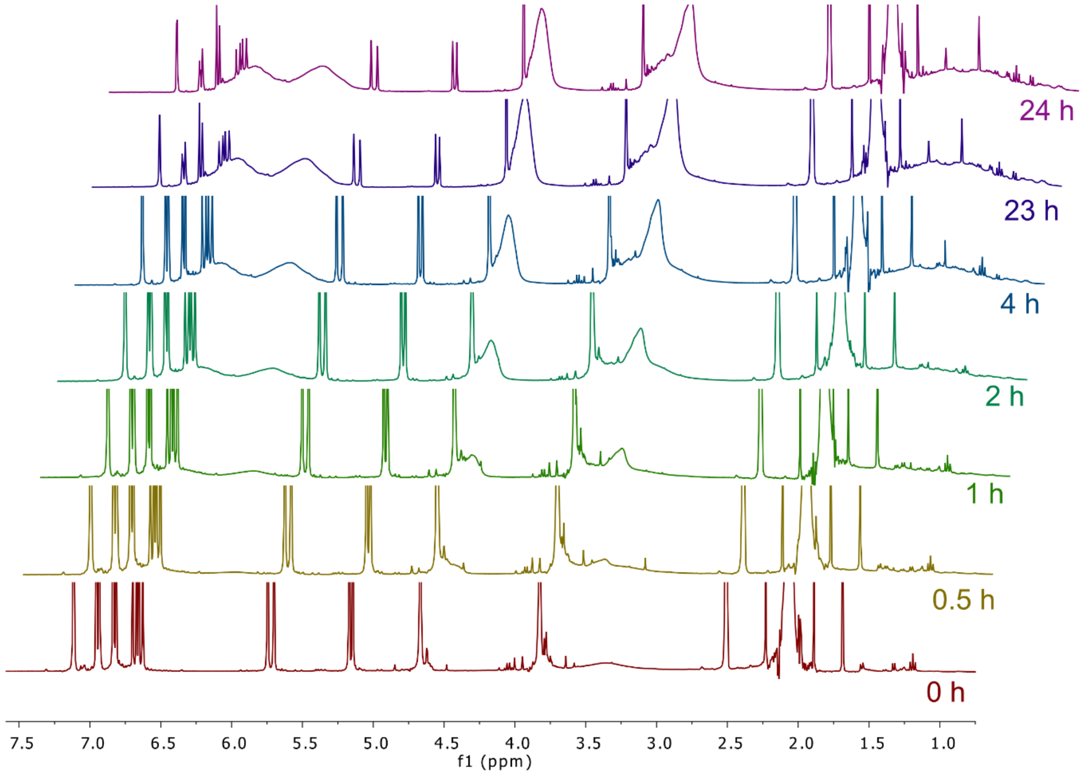

Figure S12. *^{1}H-NMR kinetics of P(MVP-AcOH) prepared by solution polymerization in DMSO-d$_6$.*

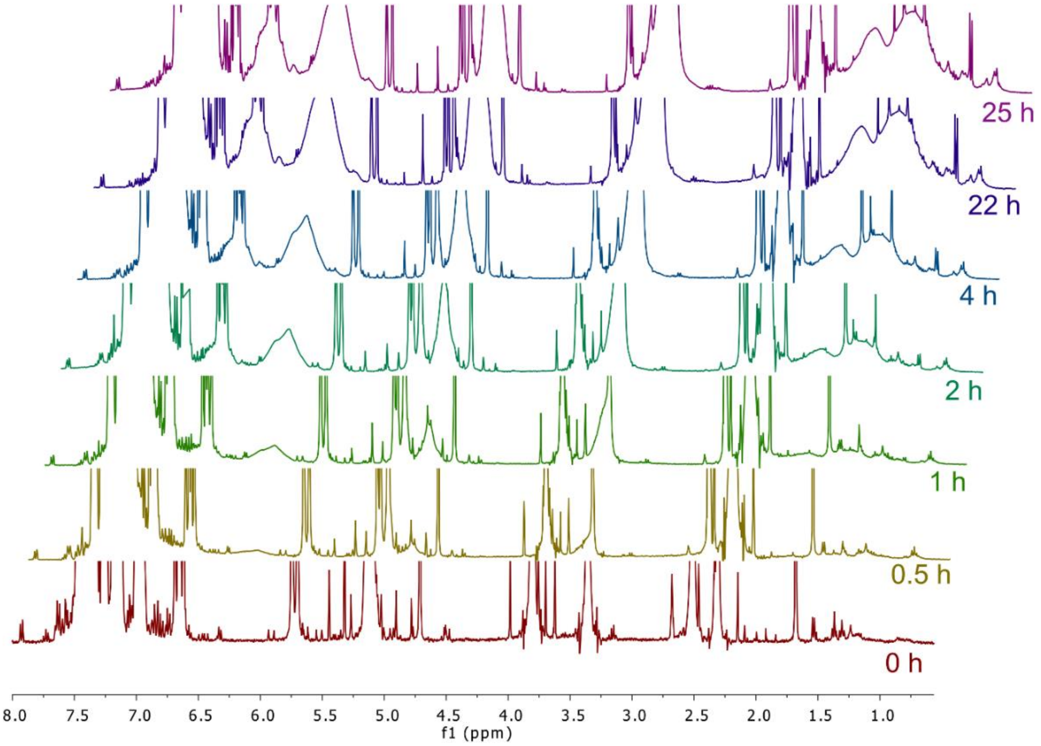

Figure S13. *^{1}H-NMR kinetics of P(MVP-Bz) prepared by solution polymerization in DMSO-d6.*

The conversion (p) of the bio-based homopolymers was calculated based on equation S1:

$$p\ (\%) = \frac{\frac{\int(methylene\ from\ polymer) - \int(methylene\ from\ monomer)}{2}}{\int(olefin\ peaks) + \frac{\int(methylene\ from\ polymer) - \int(methylene\ from\ monomer)}{2}} \times 100 \qquad \textbf{(S1)}$$

The integrals were obtained by normalizing the integrals of olefin peaks to 1000. The location of these peaks varies with the polymer and so the equation S1 becomes:

For P(MVP-AcOMe):

$$p\ (\%) = \frac{\frac{\int(4.57\ to\ 4.81\ ppm)\ -\ \int(4.76\ to\ 4.81\ ppm)}{2}}{\int(5.15\ to\ 5.22\ ppm) + \frac{\int(4.57\ to\ 4.81\ ppm) -\ \int(4.76\ to\ 4.81\ ppm)}{2}} \times 100$$

In the case of P(MVP-AcOMe), from t = 4 h, the methylene signal from monomer could barely be seen and so the integral was kept as 0.

For P(MVP-Bz):

$$p\ (\%) = \frac{\frac{\int(4.83\ to\ 5.13\ ppm)\ -\ \int(5.08\ to\ 5.12\ ppm)}{2}}{\int(5.14\ to\ 5.18\ ppm) + \frac{\int(4.83\ to\ 5.13\ ppm) -\ \int(5.08\ to\ 5.12\ ppm)}{2}} \times 100$$

For P(MVP-AcOH):

$$p\ (\%) = \frac{\frac{\int(4.38\ to\ 4.75\ ppm)\ -\ \int(4.64\ to\ 4.73\ ppm)}{2}}{\int(5.67\ to\ 5.78\ ppm) + \frac{\int(4.38\ to\ 4.75\ ppm) -\ \int(4.64\ to\ 4.73\ ppm)}{2}} \times 100$$

The conversion of each bio-based homopolymers prepared via solution polymerization is plotted against polymerization time in Figure S14.

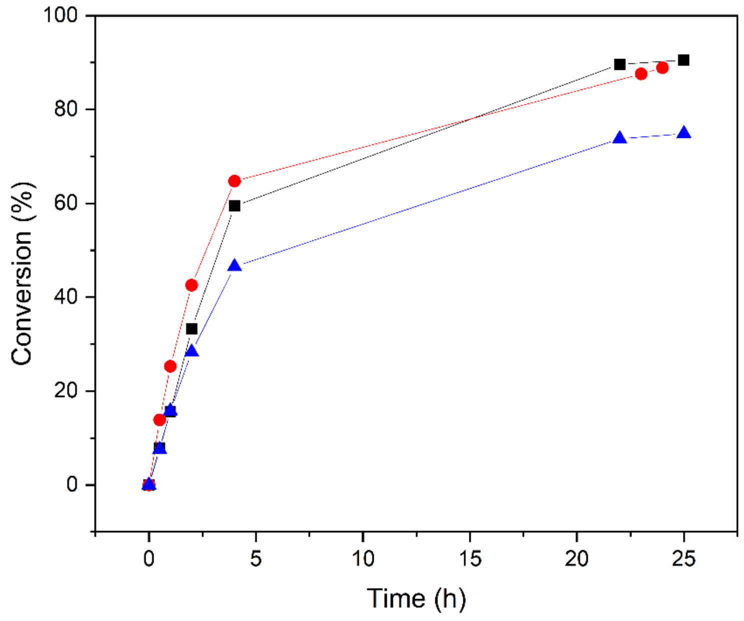

Figure S14. *Conversion plot of the solution homopolymerizations of the bio-based monomers; P(MVP-AcOMe) (black squares), P(MVP-AcOH) (red circles) and P(MVP-Bz) (blue triangles).*

4. FT-IR characterization of solution polymers

The chemical composition of the bio-based homopolymers was verified by FT-IR (Figure S15).

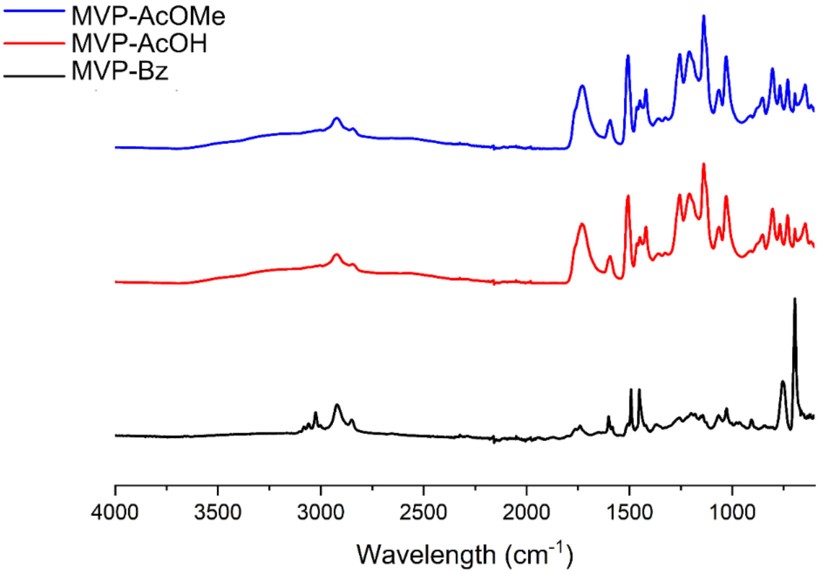

Figure S15. *FT-IR of bio-based solution homopolymers.*

5. FT-IR characterization of emulsion polymers

The chemical composition of the bio-based emulsion copolymers with styrene was verified by FT-IR (Figure S16). All of the samples look identical due to the low content of bio-based copolymer.

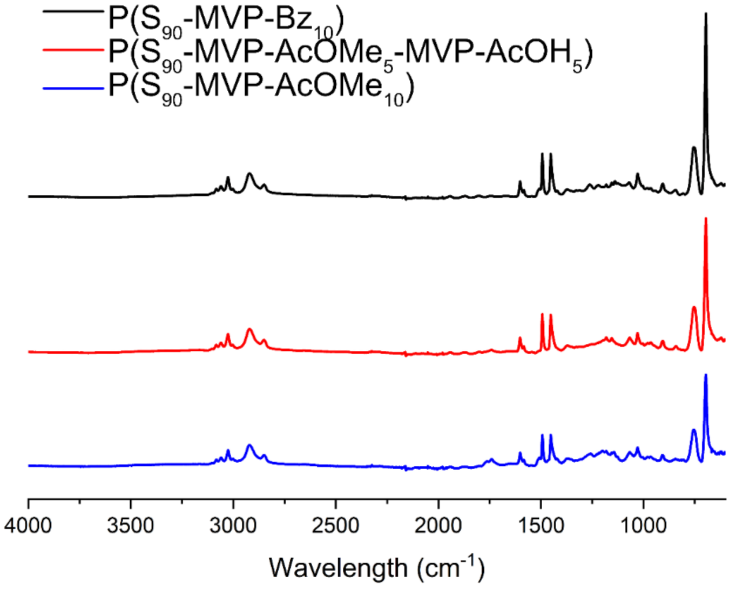

Figure S16. *FT-IR of emulsion bio-based copolymers.*

6. Emulsion copolymerization

Equation S2 is used for the calculation of the theoretical dry content ($\tau_{theor.}$) assuming a 100% conversion of monomer to polymer.

$$\tau_{theor.}\,(\%) = \left[\frac{(m_S^{initial} + m_{comonomer}^{initial})}{(m_S^{initial} + m_{comonomer}^{initial} + m_{H_2O})}\right] \cdot 100 \qquad \textbf{(S2)}$$

where $m_S^{initial}$ is the initial mass of styrene (g), $m_{comonomer}^{initial}$ is the initial mass of the bio-based comonomer used (g) and m_{H_2O} is the mass of water (g).

The conversion in emulsion polymerization was evaluated by assuming that 100% of conversion is 20 wt% of dry content. The dry content and final conversion for the emulsion copolymers are listed in Table S1.

Table S1. *Dry content and final conversion of the copolymers prepared via emulsion polymerization.*

Sample Name	Dry content (%)	Total conversion (%)
**P(S_{90}-MVP-Bz_{10}) (Batch 1)**	18.26	91.3
**P(S_{90}-MVP-Bz_{10}) (Batch 2)**	18.61	93.1
**P(S_{90}-MVP-$AcOMe_{10}$) (Batch 1)**	18.29	91.4
**P(S_{90}-MVP-$AcOMe_{10}$) (Batch 2)**	19.12	95.6
**P(S_{90}-MVP-$AcOMe_5$-MVP-$AcOH_5$) (Batch 1)**	16.18	80.9
**P(S_{90}-MVP-$AcOMe_5$-MVP-$AcOH_5$) (Batch 2)**	16.11	80.5
**PS**	19.98	99.9

The molar composition of the copolymers is obtained from ^{1}H-NMR by peak integration method from the coagulated latex dissolved in $CDCl_3$. The molar percentages are then converted to weight percentages by using the molecular weight of the comonomer.

P(S_{90}-MVP-Bz_{10}):

$$\mathrm{MVP-Bz}\ composition\ (mol\ \%) = \frac{\frac{\int methoxy\ protons}{3}}{\frac{\int aromatic\ protons}{13}} \times 100$$

$$\mathrm{MVP-Bz}\ composition\ (mol\ \%) = \frac{\frac{\int (3.54\ to\ 3.70\ ppm)}{3}}{\frac{\int (5.87\ to\ 7.58\ ppm, excluding\ solvent)}{13}} \times 100$$

$$\mathrm{MVP-Bz}\ composition\ (wt.\%) = \mathrm{MVP-Bz}\ composition\ (mol\ \%) \times 240.3$$

$$Styrene\ composition\ (wt.\%) = 100 - \ BMVB\ composition\ (wt.\%)$$

Poly(S_{90}-MVP-$AcOMe_{10}$):

$$\mathrm{MVP-AcOMe}\ composition\ (mol\ \%) = \frac{\frac{\int methylene\ protons}{2}}{\frac{\int aromatic\ protons}{8}} \times 100$$

$$\mathrm{MVP-AcOMe}\ composition\ (mol\ \%) = \frac{\frac{\int (4.49\ to\ 4.70\ ppm)}{2}}{\frac{\int (5.84\ to\ 7.58\ ppm, excluding\ solvent)}{8}} \times 100$$

$$\mathrm{MVP-AcOMe}\ composition\ (wt.\%) = MeMVPA\ composition\ (mol\ \%) \times 222.24$$

$$Styrene\ composition\ (wt.\%) = 100 - \ MeMVPA\ composition\ (wt.\%)$$

Poly(S_{90}-MVP-$AcOMe_5$-MVP-$AcOH_5$):

$$\text{ratio between MVP} - \text{AcOMe and MVP} - \text{AcOH}\ composition = \frac{\frac{\int methyle\ protons}{3}}{\frac{\int methoxy\ protons}{6}} \times 100$$

$$\text{MVP}-\text{AcOMe}\; composition\; (mol\;\%) = \frac{\frac{\int(3.75\; to\; 3.85\; ppm)}{3}}{\frac{\int(3.5\; to\; 3.7\; ppm)}{6}} \times 100$$

$$\text{MVP}-\text{AcOMe}-\text{MVP}-\text{AcOH}\; composition\; (wt.\%)$$
$$= \text{MVP}-\text{AcOMe}-\text{MVP}-\text{AcOH}\; composition\; (mol\;\%) \times 222.24$$

$$Styrene\; composition\; (wt.\%) = 100 - \text{MVP}-\text{AcOMe}-\text{MVP}-\text{AcOH}\; composition\; (wt.\%)$$

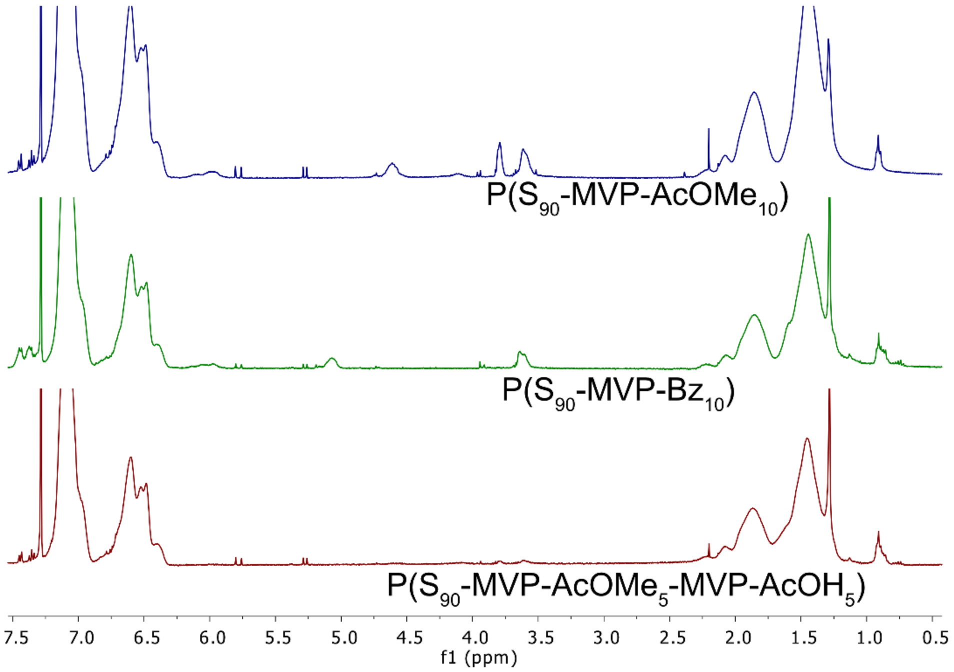

Figure S17. *^{1}H-NMR spectra of the bio-based emulsion copolymers.*

7. Digital images of the emulsion polymers

The milky appearance of the bio-based emulsions can be seen in Figure S18 top. The same samples were also heat treated at 150 °C for 2 h to remove the water and anneal them targeting a film formation (Figure S18 bottom). This could not be achieved.

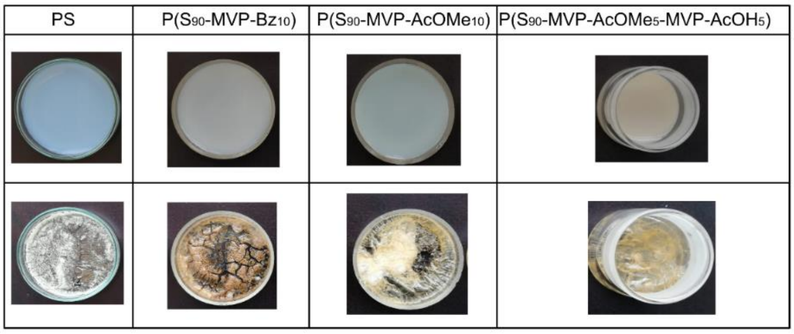

Figure S18. *Digital images of the bio-based emulsion polymers; (top) liquid dispersion before annealing and (bottom) after annealing.*

8. DLS results

The DLS results of the bio-based emulsions are listed in Table S2.

Table S2. *DLS results of bio-based emulsions.*

**Sample Name**	D_H (nm)	PdI	ζ (mW)
**PS**	58.4±0.3	0.07±0.01	-65±5
**P(S_{90}-MVP-$AcOMe_{10}$)**	52±1	0.08±0.01	-49±1
**P(S_{90}-MVP-Bz_{10})**	52±2	0.15±0.02	-51±2
**P(S_{90}-MVP-$AcOMe_5$-MVP-$AcOH_5$)**	80±23*	0.54±0.04*	-58±3*

** multiple peaks were observed in DLS data (Table S3)*

Table S3. *DLS results of P(S_{90}-MVP-$AcOMe_5$-MVP-$AcOH_5$).*

**Sample Name**	D_H^1 (nm)[a]	A^1 (%)[b]	D_H^2 (nm)[a]	A^2 (%)[b]	D_H^3 (nm)[a]	A^3 (%)[b]
P(S_{90}-MVP-$AcOMeA_5$-MVP-$AcOH_5$)	44.1±0.3	62.0±1.8	713.9±142.0	37.1±0.6	4808	2.7

[a] *peak of the hydrodynamic diameter obtained from DLS.* [b] *percentage of the peak area obtained from DLS.*

9. TGA of solution and emulsion polymers

The first weight derivative obtained from TGA is plotted against temperature for the solution (Figure S19a) and emulsion polymers (Figure S19b).

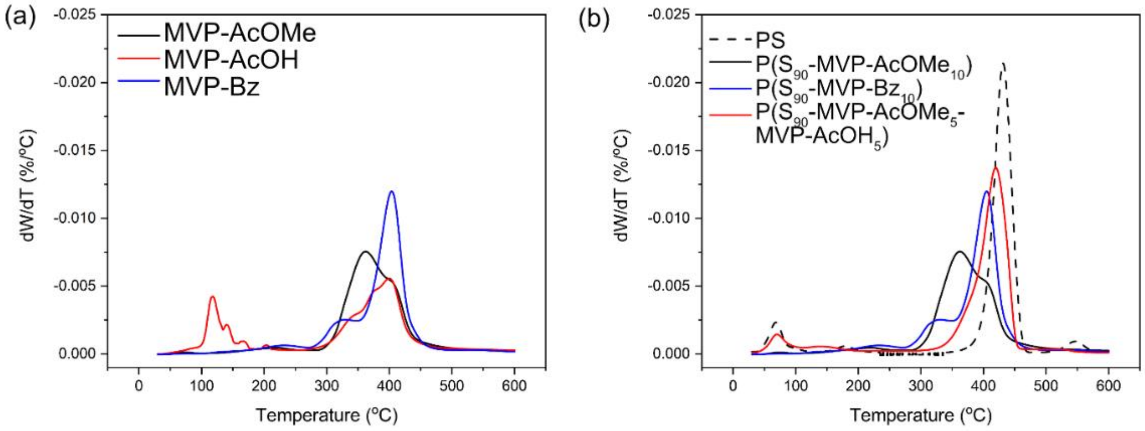

Figure S19. *First derivative from TGA for solution (a) and emulsion (b) polymers.*

The ash content (AC) obtained at 600 °C from TGA and the temperature (T_m) and enthalpy of melting ($\Delta H_{melting}$) obtained from DSC are listed in Table S4.

Table S4. *Ash content for the solution and emulsion polymers.*

Sample Name	Type [a]	T_m (°C)[c]	$\Delta H_{melting}$ (J/g)[c]	AC (%)[b]
**MVP-AcOMe**	N.A.	N.A.	N.A.	N.A.
**MVP-AcOH**	N.A.	109±1	157±2	N.A.
**MVP-Bz**	N.A.	51±1	76±8	N.A.
**P(MVP-AcOMe)**	S	----	----	24±2
**P(MVP-AcOH)**	S	----	----	26±4
**P(MVP-Bz)**	S	----	----	17±1
**PS**	E	----	----	7.1±0.2
**P(S_{90}-MVP-AcOMe$_{10}$)**	E	----	----	8±1
**P(S_{90}-MVP-Bz$_{10}$)**	E	----	----	9±1
**P(S_{90}-MVP-AcOMe$_5$-MVP-AcOH$_5$)**	E	----	----	12±1

[a] *Type of polymerization used, i.e., solution (S) or emulsion (E).* [b] *Ash content was determined from the weight percent at 600 °C from TGA.*

10. DSC traces of the thermoplastic materials

The DSC traces of the MVP-based monomers used for thermoplastic applications is shown in Figure S20. The first heating is shown where a melting peak is observed for MVP-Bz and MVP-AcOH.

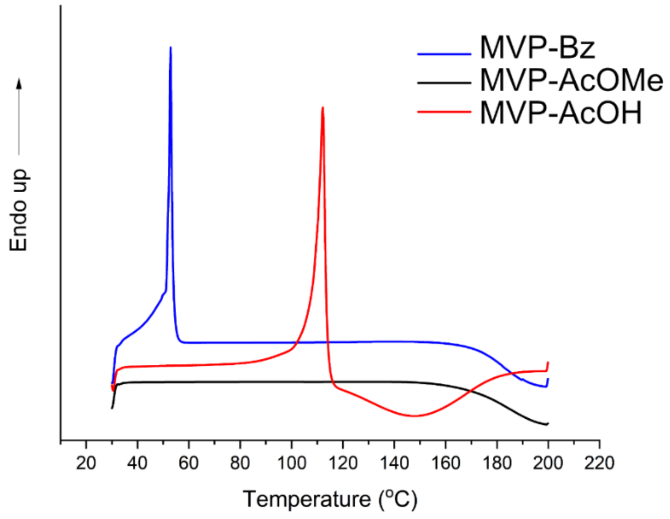

Figure S20. *DSC traces of the MVP-based monomers.*

The DSC traces of the bio-based polymers prepared via solution polymerization are shown in Figure S21.

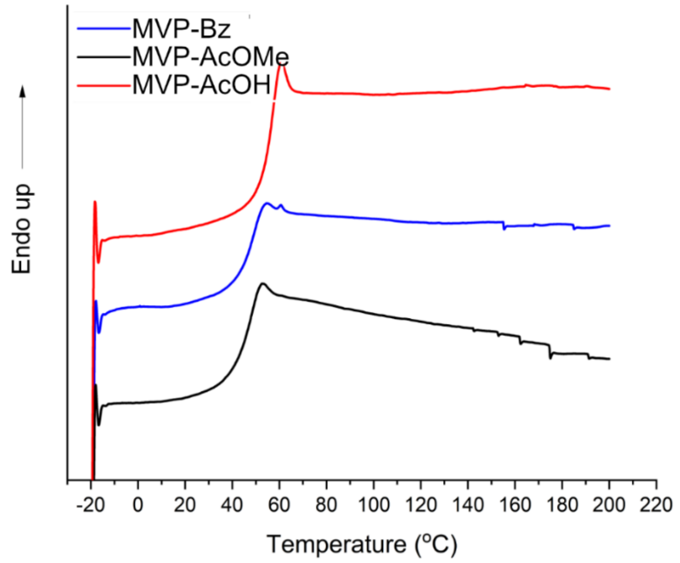

Figure S21. *DSC traces of the MVP-based polymers obtained via solution polymerization.*

The DSC traces of the bio-based polymers prepared via emulsion polymerization are shown in Figure S22.

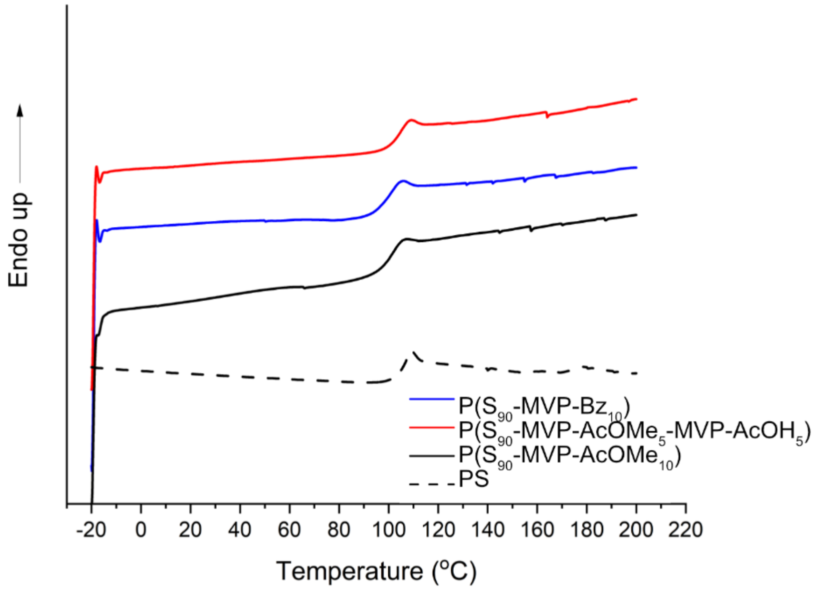

Figure S22. *DSC traces of the MVP-based polymers obtained via emulsion copolymerization with styrene.*

11. **Glass transition temperature calculation**

The theoretical glass transition temperature ($T_g^{theor.}$) of the copolymers prepared via emulsion polymerization was calculated by using the Flory-Fox equation S3 [58]:

$$\frac{1}{T_g^{theor.}} = \frac{w_1}{T_g^1} + \frac{w_2}{T_g^2} \qquad \textbf{(S3)}$$

Where $T_g^{theor.}$ is the theoretical T_g of the copolymer, T_g^1 is the T_g of homopolymer of PS, T_g^2 is the T_g of homopolymer of the bio-based comonomer (P(MVP-AcOMe), P(MVP-AcOH) or P(MVP-Bz)), w_1 is the weight fraction of styrene and w_2 is the weight fraction of the bio-based comonomers. In equation S3, all temperature values used were converted into Kelvin.

The values for the bio-based comonomers and the PS homopolymer are listed in Table 2. According to equation S3, the $T_g^{theor.}$ for the copolymers are: 98.8 °C, 100.0 °C and 99.5 °C for P(S_{90}-MVP-$AcOMe_{10}$), P(S_{90}-MVP-Bz_{10}) and P(S_{90}-MVP-$AcOMe_5$-MVP-$AcOH_5$), respectively.

PART B

1. Amounts of reagents for the synthesis of DVB-like monomers

The analytical amounts used for the preparation of the distyrene monomers are listed in Table S5.

Table S5. *Amounts used for the synthesis of crosslinkers.*

Name of crosslinker	MVP (g, mmol)	Acetone (mL)	TBAI (g, mmol)	1,6-DBH (g, mmol)	1,10-DBD (g, mmol)
**1,6-MVP**	6.5, 43	153	0.229, 0.015	4.53, 18.6	----
**1,10-MVP**	6.5, 43	153	0.185, 0.015	----	4.50, 15.0

2. Amounts of reagents for thermoset synthesis

The analytical amounts used for the preparation of the thermally cured films are listed in Table S6.

Table S6. *Amounts used for the preparation of thermoset films.*

Name of thermoset film	1,3-MVP (g, mmol)	1,6-MVP (g, mmol)	1,10-MVP (g, mmol)	3T (g, mmol)	4T (mg, mmol)
**X(1,3-MVP–3T)**	0.14, 0.39	----	----	0.11, 0.26	----
**X(1,3-MVP–4T)**	0.14, 0.39	----	----	----	0.10, 0.20
**X(1,6-MVP–3T)**	----	0.14, 0.35	----	0.09, 0.24	----
**X(1,6-MVP–4T)**	----	0.14, 0.35	----	----	0.08, 0.18
**X(1,10-MVP–3T)**	----	----	0.14, 0.31	0.08, 0.21	----
**X(1,10-MVP–4T)**	----	----	0.14, 0.31	----	0.07, 0.15

3. NMR characterization of DVB-like monomers

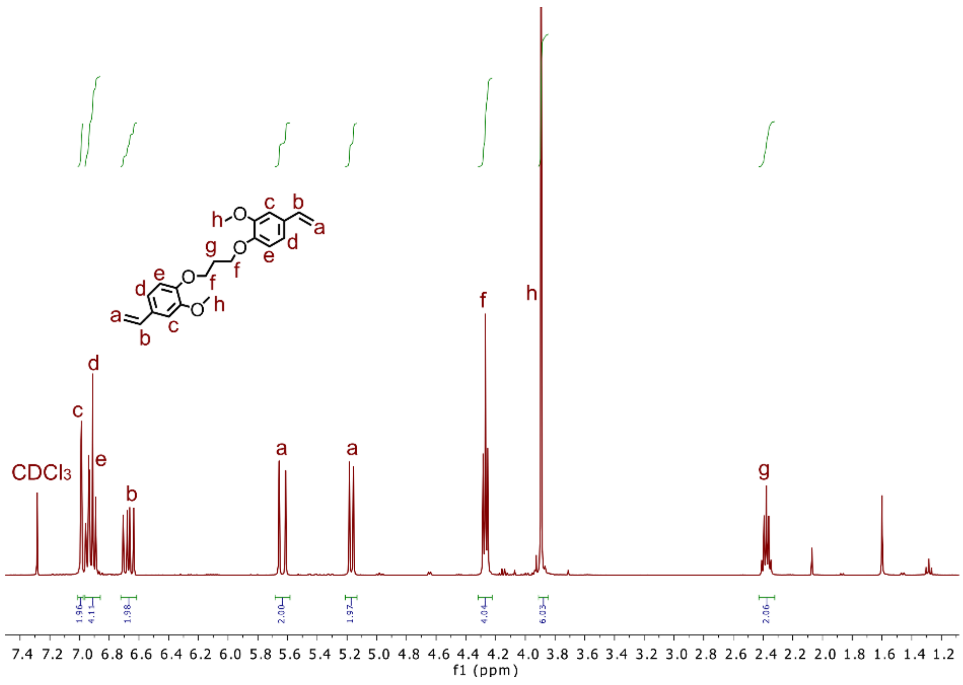

Figure S23. *^{1}H-NMR of 1,3-MVP in CDCl$_3$.*

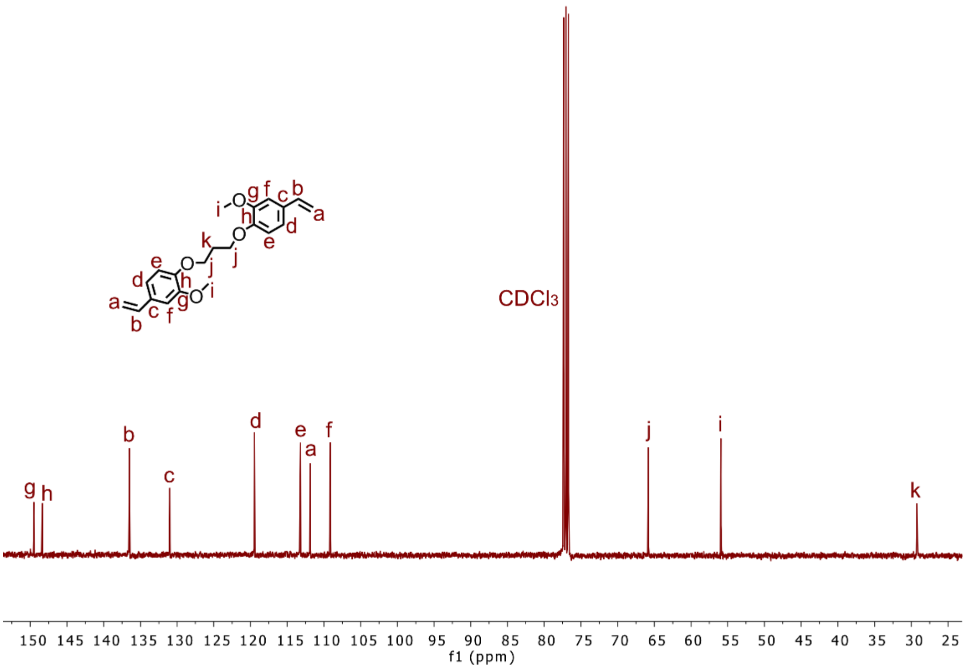

Figure S24. *^{13}C-NMR of 1,3-MVP in CDCl$_3$.*

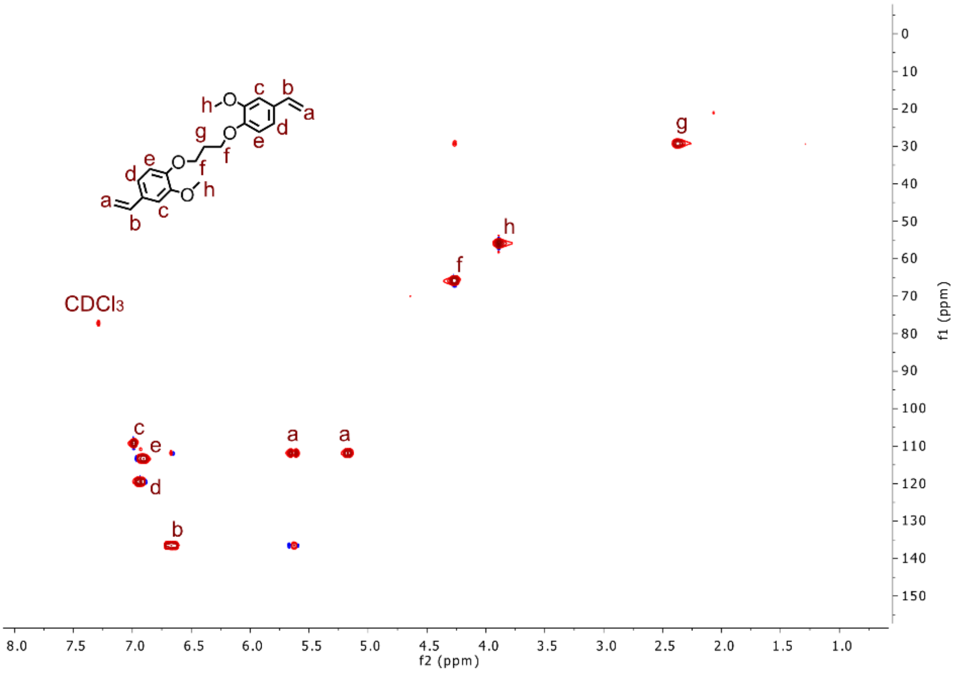

Figure S25. *HSQC of 1,3-MVP in CDCl₃.*

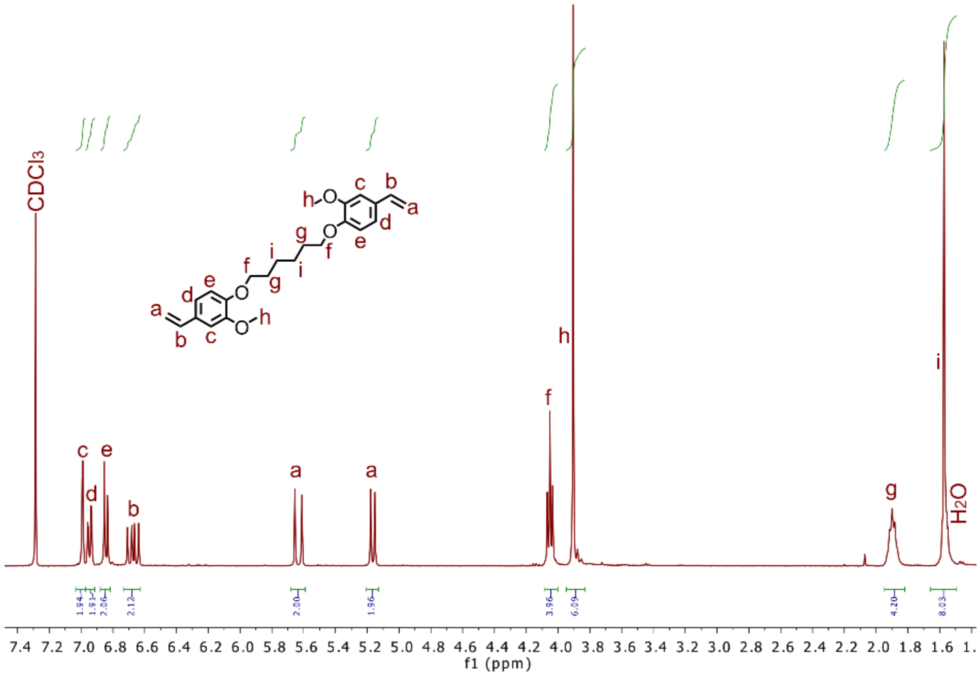

Figure S26. *^{1}H-NMR of 1,6-MVP in CDCl₃.*

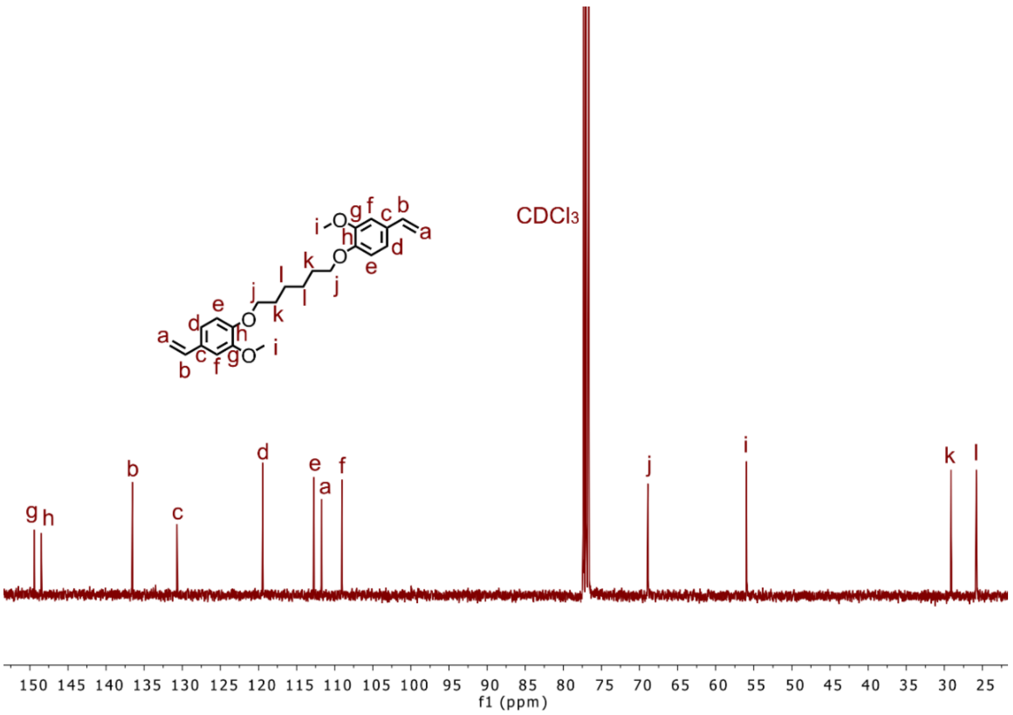

Figure S27. *^{13}C-NMR of 1,6-MVP in $CDCl_3$.*

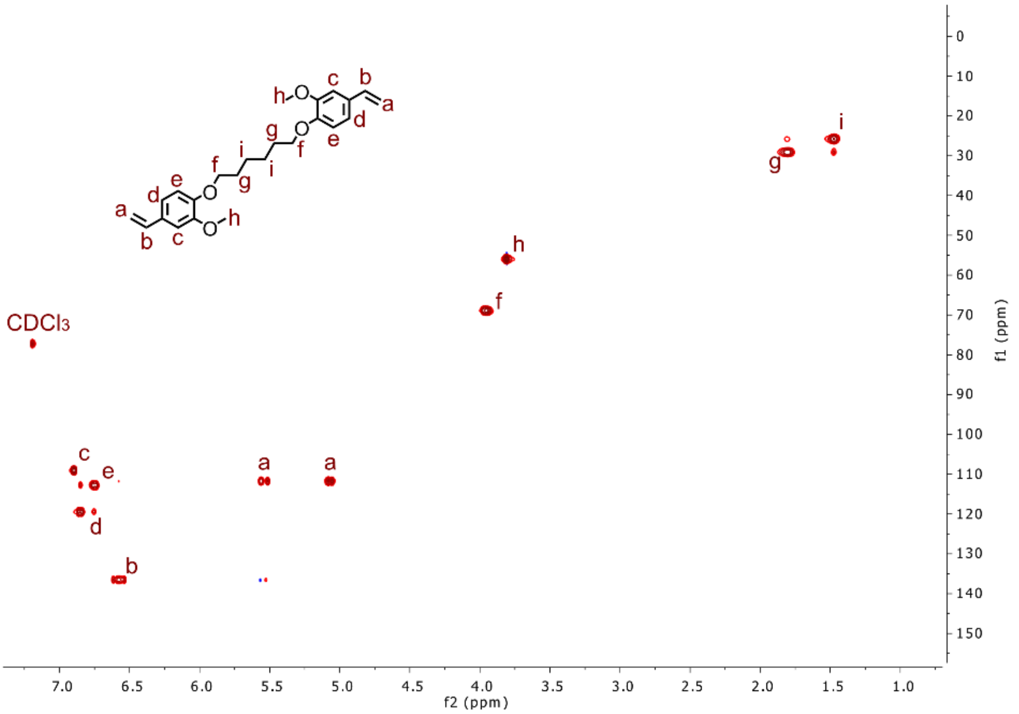

Figure S28. *HSQC of 1,6-MVP in $CDCl_3$.*

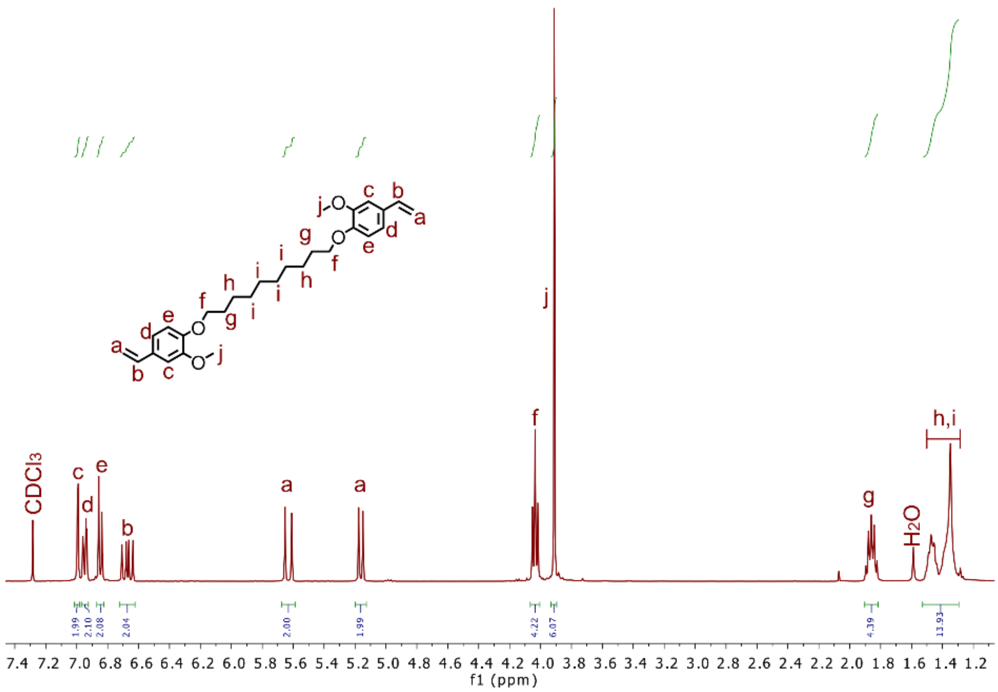

Figure S29. *^{1}H-NMR of 1,10-MVP in $CDCl_3$.*

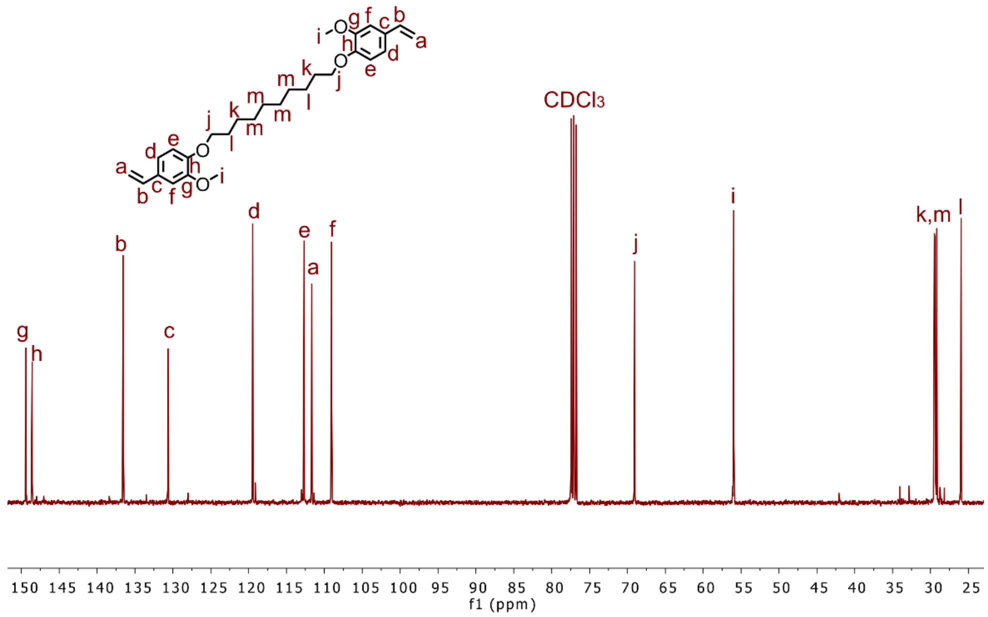

Figure S30. *^{13}C-NMR of 1,10-MVP in $CDCl_3$.*

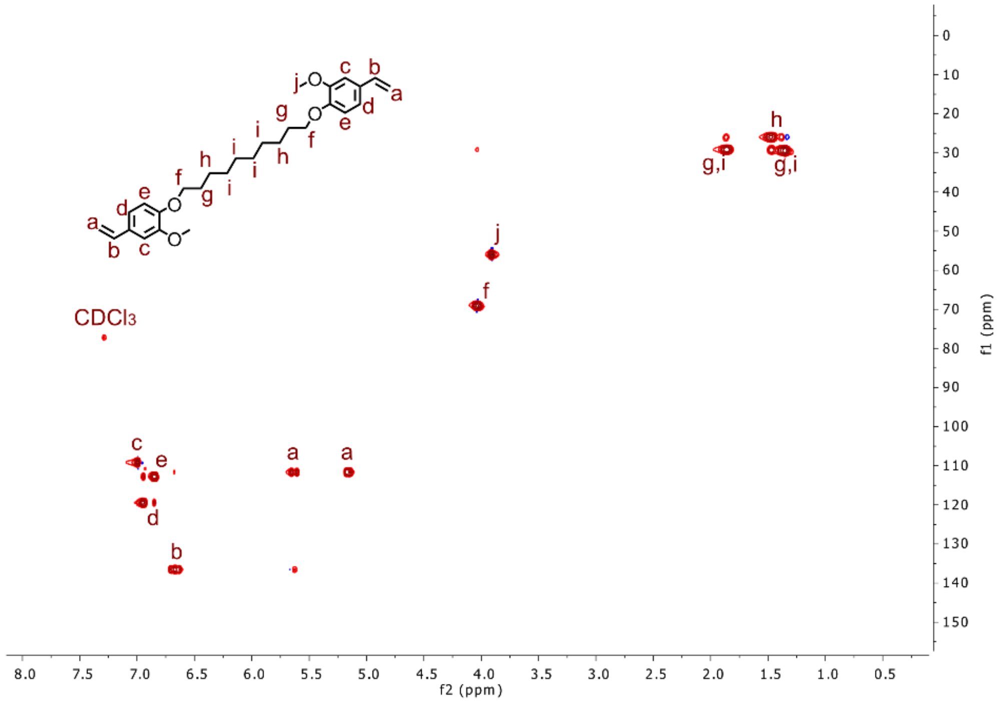

Figure S31. *HSQC of 1,10-MVP in $CDCl_3$.*

4. FT-IR characterization of DVB-like monomers for thermosetting applications

The chemical composition of the bio-based monomers used to prepare distyrene monomers for thermosetting applications was verified by FT-IR (Figure S32). The 1,6-MVP and 1,10-MVP exhibit two pronounced peaks around 2800 cm^{-1} which is attributed to the longer aliphatic chain connecting the two MVP units. The same peak is less pronounced in 1,3-MVP due to the smaller length. Additionally, the absence of the phenol peak observed on MVP indicates the successful modification.

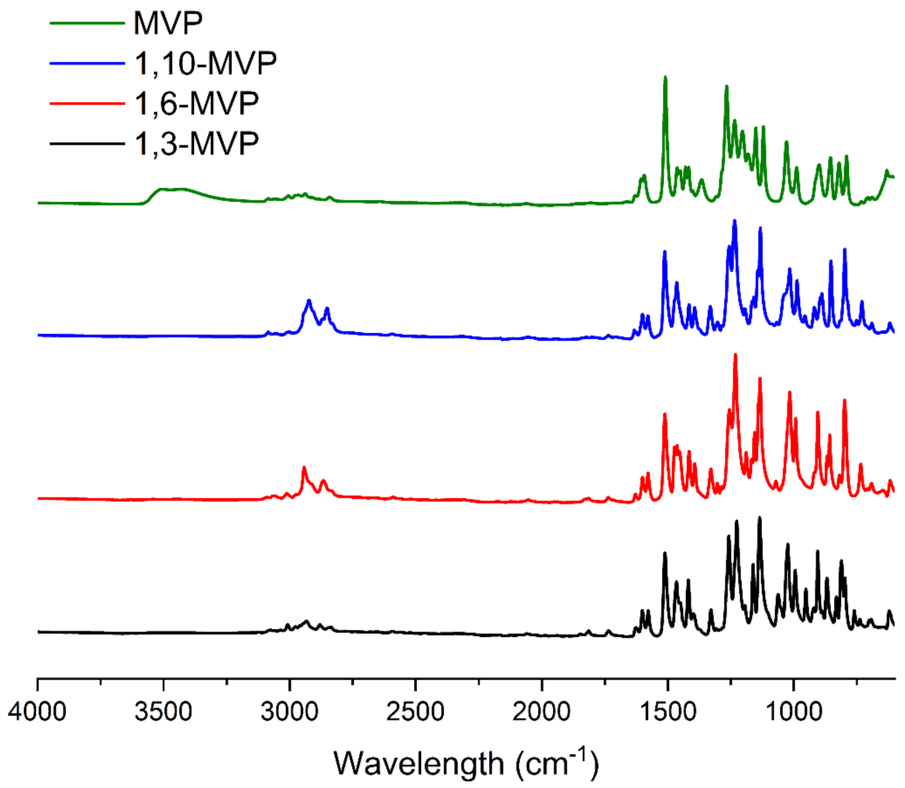

Figure S32. *FT-IR of bio-based monomers for thermosetting applications.*

5. FT-IR characterization of DVB-based thermosets

The chemical composition of the final bio-based thermosets was studied by FT-IR (Figure S33). All samples exhibit the same peaks. Also, the absence of any -SH peak, which typically appear between 2600 and 2550 cm^{-1} is a good indication that all thiol units have reacted.

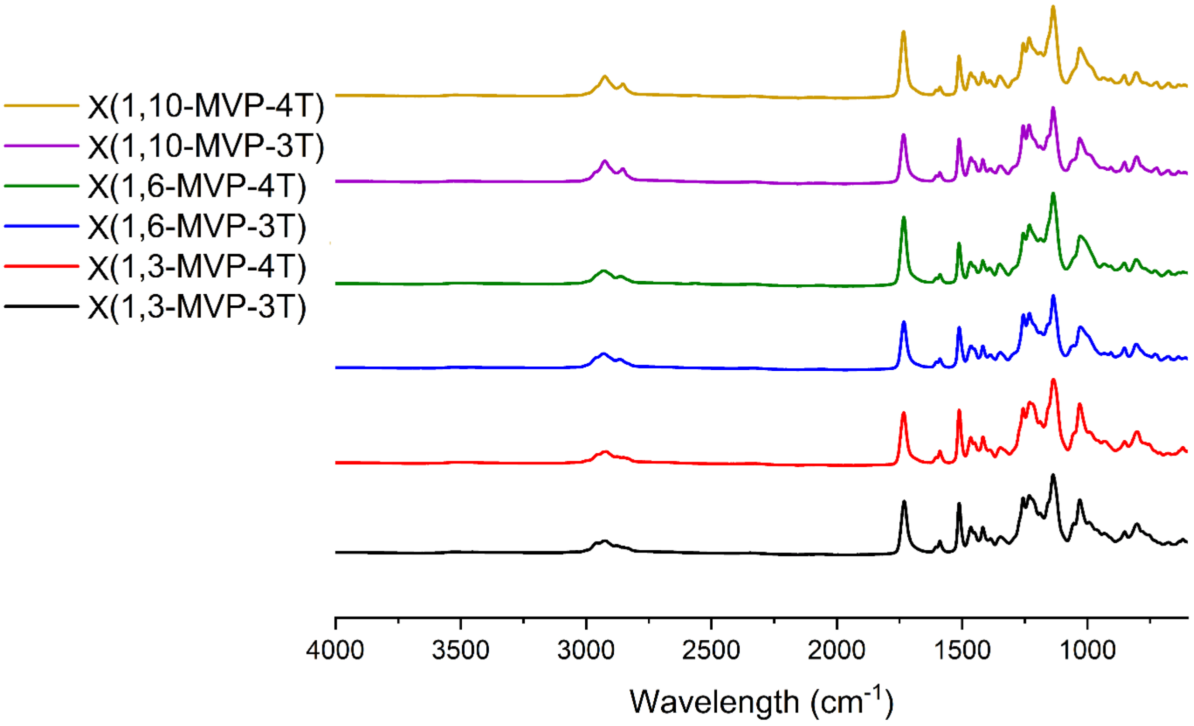

Figure S33. *FT-IR of bio-based thermosets based on MVP-derived distyrene monomers.*

6. Digital images of the thermosets

The colour and rectangular shape of the thermosets can be seen in Figure S34.

X(1,3-MVP-3T)	X(1,3-MVP-4T)	X(1,6-MVP-3T)	X(1,6-MVP-4T)	X(1,10-MVP-3T)	X(1,10-MVP-4T)
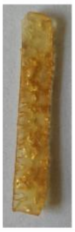	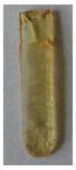	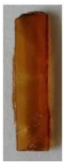	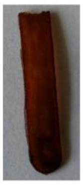	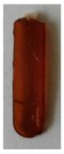	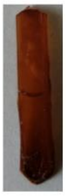

Figure S34. *Digital images of the bio-based thermosets after DMA characterization.*

7. TGA results of thermosets

The thermograms of the thermosets can be found in Figure S35.

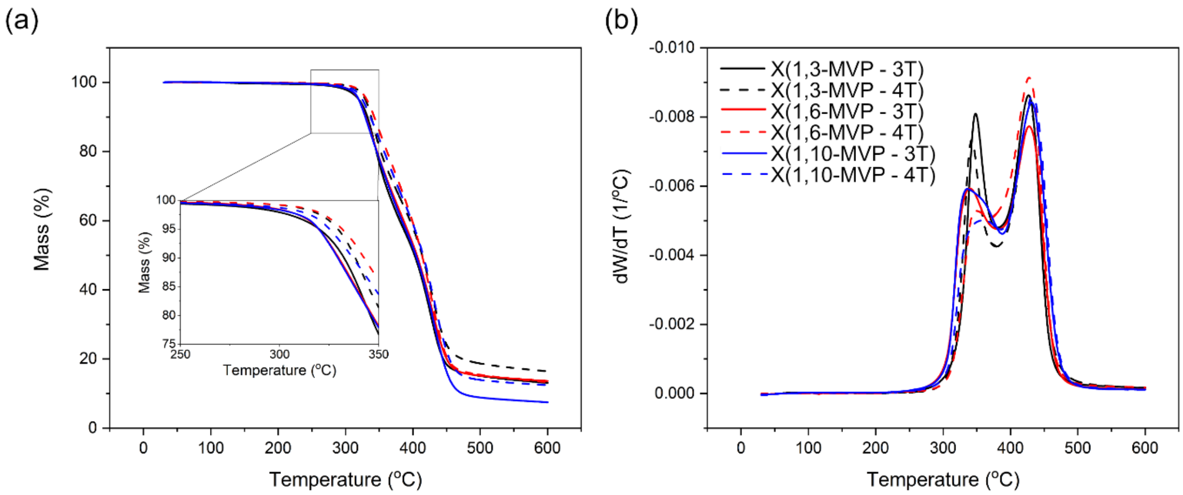

Figure S35. *TGA thermograms of the thermosets (a) and the first derivative of the weight (b). The inlet in (a) shows a magnification of the onset of degradation.*

The AC of the thermosets obtained at 600 °C from TGA is listed in Table S7.

Table S7. *Ash content of the thermosets.*

Sample Name	T_{deg} (°C)[a]	AC (%)[b]
**X(1,3-MVP–3T)**	325±8	13.8±1.0
**X(1,3-MVP–4T)**	332±4	16.7±0.3
**X(1,6-MVP–3T)**	322±4	13.3±0.6
**X(1,6-MVP–4T)**	333±3	13.3±0.1
**X(1,10-MVP–3T)**	321±2	9.1±2.2
**X(1,10-MVP–4T)**	325±1	11.9±0.8

[a] *Degradation temperature determined from the 5 wt% loss in mass.* [b] *Ash content was determined from the weight percent at 600 °C from TGA.*

8. DSC traces of thermosets

The DSC traces of the MVP-based monomers used for thermosetting applications is shown in Figure S36. The DSC traces are from the first heating cycle in order to study the melting temperature of the DVB monomers.

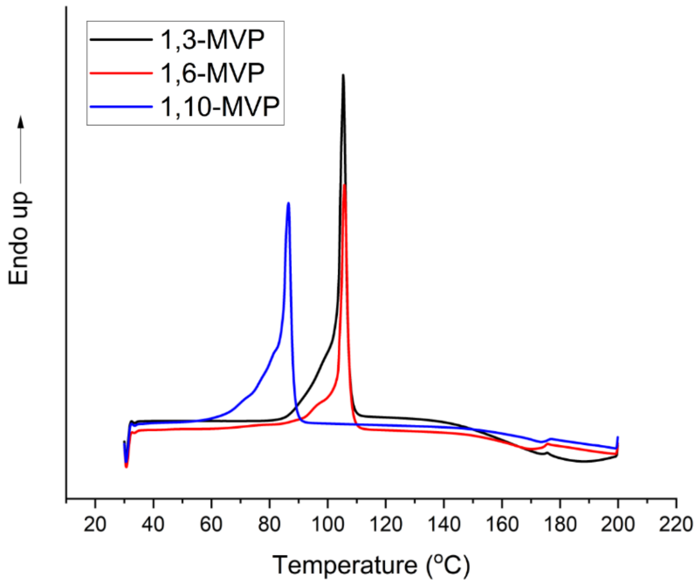

Figure S36. *DSC traces of the DVB monomers.*

The thermal curing reaction between the DVB-lime monomers and the thiol crosslinkers was also studied by DSC (Figure S37). The maximum curing peaks for each combination of thiol crosslinker and DVB-like monomers is listed in Table S8.

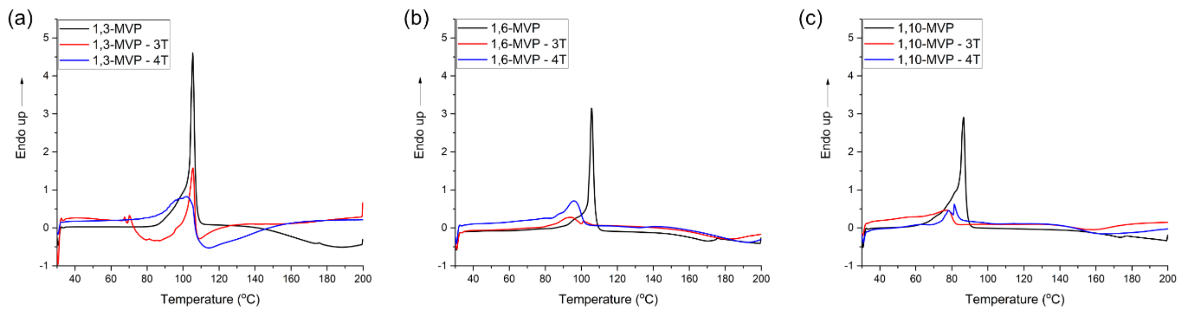

Figure S37 – *Thermal curing studied in situ during DSC analysis. The cases of (a) 1,3-MVP, (b) 1,6-MVP and (c) 1,10-MVP. The samples with 3T (red graph) and 4T (blue graph) are also shown.*

Table S8 – *Maximum curing peak extrapolated from the DSC traces of Figure S37.*

Mixture combination	T_{curing}^{max} (°C)
**1,3-MVP + 3T**	109
**1,3-MVP + 4T**	114
**1,6-MVP + 3T**	183
**1,6-MVP + 4T**	188
**1,10-MVP + 3T**	158
**1,10-MVP + 4T**	164

The DSC traces of the thermally cured thermosets are shown in Figure S37.

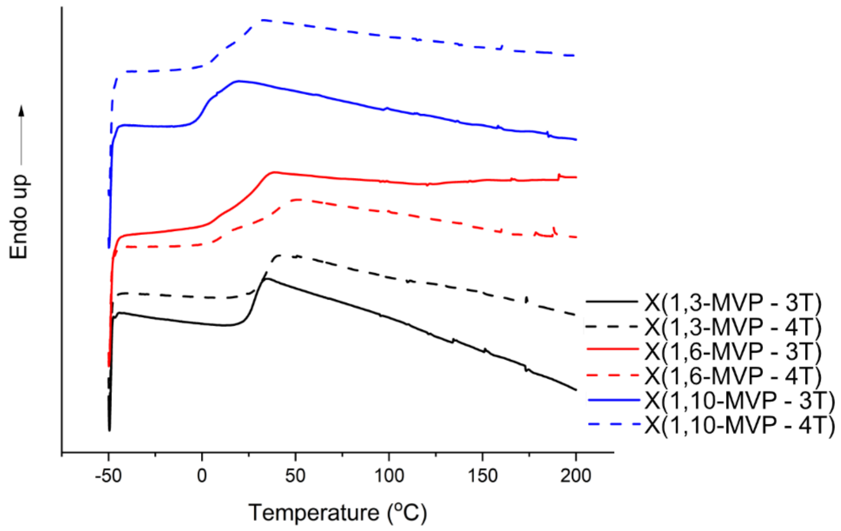

Figure S38. *DSC traces of the thermally crosslinked thermosets.*

Table S9 – *Melting temperature and enthalpy of melting of the DVB-like monomers obtained from DSC.*

**Sample Name**	T_m (°C)[b]	$\Delta H_{melting}$ (J/g)
**1,3-MVP**	105±1	127±1
**1,6-MVP**	104±1	79±3
**1,10-MVP**	85.8±0.2	105±2

9. Crosslinking density and gel content calculation

The crosslinking density (v_e) was calculated using equation S4. [54]

$$v_e = \frac{E'_{80\,°C}}{3 \cdot R \cdot T} \qquad \textbf{(S4)}$$

where $E'_{80\,°C}$ is the storage modulus at 80 °C taken as the plateau modulus after the glass transition for all samples, R is the gas constant (8.314 J/mol K) and T is the temperature at 80 °C in Kelvin (353.15 K).

Gel content was calculated using equation S5.[1] Briefly, thermoset films of approximately 30 mg were put in a vial containing 10 mL of DCM and left for 24 h. Then, they were removed and dried.

$$Gel\ content\ (\%) = \frac{m_{dried}}{m_{initial}} \cdot 100\% \qquad \textbf{(S5)}$$

where $m_{initial}$ is the initial mass of the thermosets and m_{dried} is the final dried mass after they were dipped in DCM.

The reason DCM was used is based on previous experiments in which DCM solubilized both the DVB-like monomers and the thiol crosslinkers.

10. Chemical stability and polarity of the thermosets

The chemical stability of the thermosetting films was studied by immersing approximately 10 mg of a film in 10 mL of aqueous solution or organic solvent (Figure S39). Also, the polarity of the thermosets was studied by measuring the contact angle against water (Table S10).

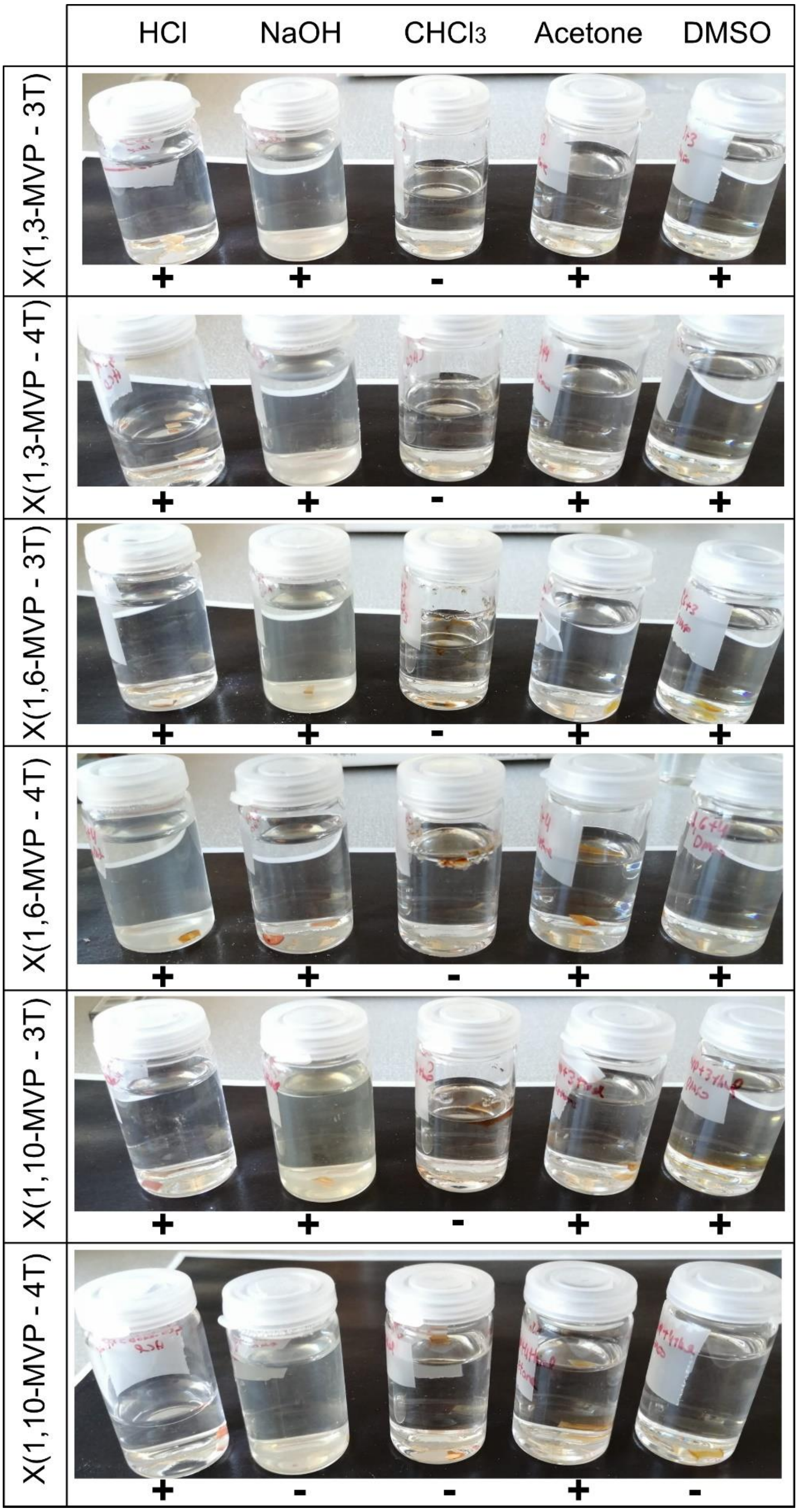

Figure S39. *Digital images of the chemical stability test of the thermosets. From left to right: HCl 1M, NaOH 1M, $CHCl_3$, acetone and DMSO. "+" mark means that the thermoset was intact and "-" means that the thermoset was broken down in smaller pieces after 7 days.*

Table S10. *Chemical stability of thermosetting films.*

Sample Name	CA (°)[a]
**XP(1,3-MVP–3T)**	77±4
**X(1,3-MVP–4T)**	75±7
**X(1,6-MVP–3T)**	73±6
**X(1,6-MVP–4T)**	75±8
**X(1,10-MVP–3T)**	72±4
**X(1,10-MVP–4T)**	78±10

[a] *Contact angle against water.*

11. Environmental impact and comparison

In this study three different approaches were used to evaluate the impact our process and the final products had on the environment, i.e. atom efficiency (AE), E-factor and bio-based carbon content (BBCC).

Atom efficiency (AE) can be calculated by using equation S6 [38]:

$$AE = \frac{M_w^{product}}{\sum M_w^{starting\ materials}} * 100 \qquad \textbf{(S6)}$$

where $M_w^{product}$ is the molecular weight of product, i.e. 222.24 g/mol (MVP-AcOMe), 208.21 g/mol (MVP-AcOH), 240.30 g/mol (MVP-Bz), 340.42 g/mol (1,3-MVP), 382.50 g/mol (1,6-MVP) and 438.61 g/mol (1,10-MVP) and $M_w^{starting\ materials}$ is the molecular weight of reagents used, i.e. 184.18 g/mol (FA), 150.18 g/mol (MVP), 152.97 g/mol (MeBrAc), 23.95 g/mol (LiOH), 171.04 g/mol (BzBr), 201.89 g/mol (1,3-DBP), 243.94 g/mol (1,6-DBH) and 300.07 g/mol (1,10-DBD).

The environmental factor (E) can be calculated by using equation S7 [39,40]:

$$E = \frac{\sum m_{reagents} + \sum m_{solvents} - \sum m_{product}}{\sum m_{product}} \quad \textbf{(S7)}$$

where $m_{product}$ is the mass of product, $m_{product}$ is the mass of the solvents and $m_{reagents}$ is the mass of all reagents used to produce the desired product.

Bio-based carbon content (BBCC) can be calculated by using equation S8 [41]:

$$BBCC = \frac{N_{bio-based\ carbon}^{product}}{Total\ N_{carbon}} * 100 \quad \textbf{(S8)}$$

where $N_{bio-based\ barbon}^{product}$ is the number of bio-based carbons in the product and $Total\ N_{carbon}$ is the total number of carbons in the product.

The values of each parameter are listed in Table S11 for all bio-based monomers synthesized in this study.

Table S11. *Bio-based characteristics of the synthesized bio-monomers.*

Sample Name	AE (%)	E	BBCC (%)
**MVP**	77.3	20.4	100.0
**MVP-AcOMe**	73.3	18.2	75.0
**MVP-AcOH**	63.7	134.7	81.8
**MVP-Bz**	74.8	18.6	56.3
**1,3-MVP**	62.5	63.0	85.7
**1,6-MVP**	70.2	72.6	75.0
**1,10-MVP**	73.0	58.7	64.3

A selection of the bio-based characteristics of bio-based monomers derived from lignin that can undergo radical polymerization is listed in Table S12.

Table S12. *Bio-based characteristics of a selection of lignin-derived monomers containing a functional double bond.*

Chemical Name	Derived from	AE (%)	E	BBCC (%)
**3-methoxy-4-(oxiran-2-ylmethoxy)benzyl methacrylate [42]**	Vanillin	75-88	9-17	53.3
**1-(5'-(dodec-11-enoyl)-2',6-dihydroxy-3',5-dimethoxy-[1,1'-biphenyl]-3-yl)tridec-12-en-1-one[42]**	Vanillin	94	36.2	41.0
**4-(4-(methacryloyloxy)-2-pentadecylbenzyl)-2-methoxyphenyl methacrylate [42]**	Vanillin	89	10	21.6
**4-(dec-9-en-1-yloxy)-3-methoxybenzyl (3-methoxy-4-(undec-10-en-1-yloxy)benzyl) adipate [42]**	Vanillin	79-92	0.5-2.5	37.2
**1,4-bis((4-allyl-2-methoxyphenoxy)methyl)benzene [41]**	Eugenol	N.A.	N.A.	70.2

Additionally, the chemical composition of the bio-based monomers synthesized in this study was further compared to other commercial fossil-based monomers by using the Van Krevelen diagram (Figure S39).[37]

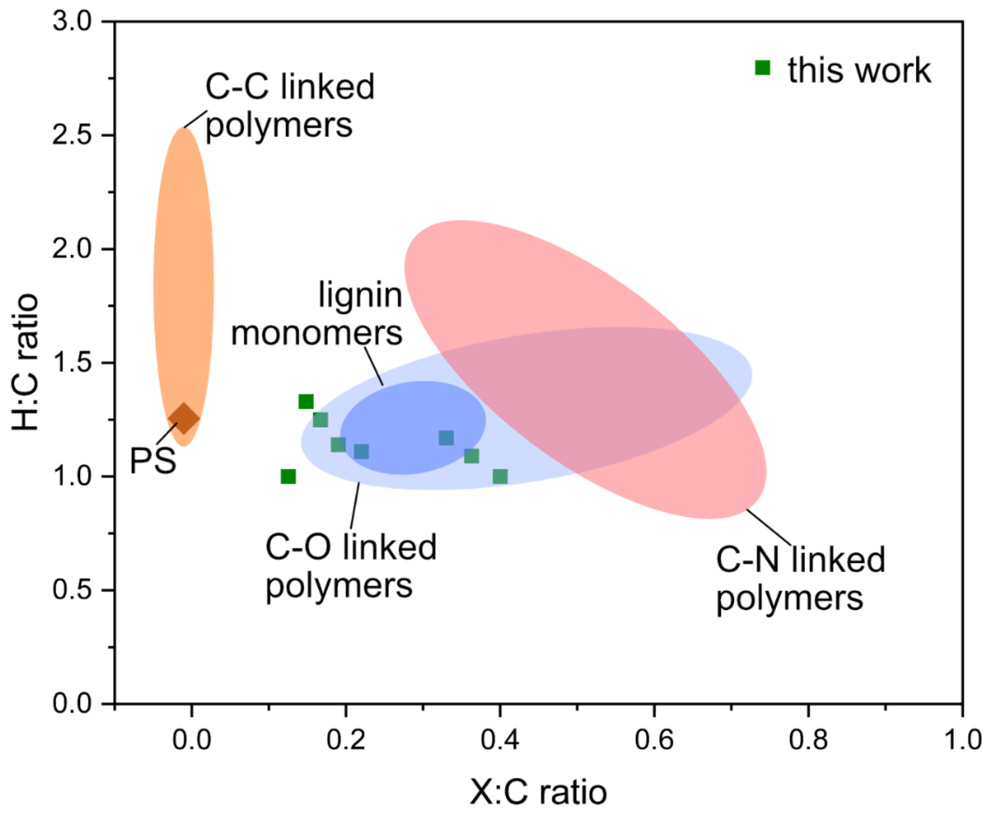

Figure S39. *Van Krevelen diagram for commercially available fossil-based and bio-based monomers/polymers in comparison with the bio-based monomers presented in this work; The results from this work coincide with other lignin monomers and C-O linked polymers in the literature. The regions selected for C-C, C-O, C-N, PS and lignin monomers were adapted from ref*[2]*.*
